# Outcomes of Robot-Assisted Surgery in Rectal Cancer Compared with Open and Laparoscopic Surgery

**DOI:** 10.3390/cancers15030839

**Published:** 2023-01-29

**Authors:** Elias Khajeh, Ehsan Aminizadeh, Arash Dooghaie Moghadam, Rajan Nikbakhsh, Gil Goncalves, Carlos Carvalho, Amjad Parvaiz, Yakup Kulu, Arianeb Mehrabi

**Affiliations:** 1 Department of General, Visceral, and Transplantation Surgery, Heidelberg University Hospital, 69121 Heidelberg, Germany; 2 Digestive Unit, Department of Surgery, Champalimaud Foundation, 1400-038 Lisbon, Portugal; 3 Digestive Unit, Department of Oncology, Champalimaud Foundation, 1400-038 Lisbon, Portugal

**Keywords:** rectal cancer, total mesorectal excision, rectal resection, costs, laparoscopic surgery, robotic surgery, systematic review, meta-analysis

## Abstract

**Simple Summary:**

Surgery is the mainstay treatment for patients with rectal cancer. Open and laparoscopic surgeries have been used for many years, but robot-assisted surgery has been recently developed and is expanding rapidly. We compared the results of robotic rectal surgery with open and laparoscopic methods. We found that robotic surgery has better outcomes than open surgery in bleeding, infection, hospital stay, and complete tumor resection. Similarly, robotic surgery did better than the laparoscopic approach in bleeding, need for reoperation, and complete tumor resection. Robotic surgery, however, incurred longer operation times and higher costs than open and laparoscopic surgery did.

**Abstract:**

With increasing trends for the adoption of robotic surgery, many centers are considering changing their practices from open or laparoscopic to robot-assisted surgery for rectal cancer. We compared the outcomes of robot-assisted rectal resection with those of open and laparoscopic surgery. We searched Medline, Web of Science, and CENTRAL databases until October 2022. All randomized controlled trials (RCTs) and prospective studies comparing robotic surgery with open or laparoscopic rectal resection were included. Fifteen RCTs and 11 prospective studies involving 6922 patients were included. The meta-analysis revealed that robotic surgery has lower blood loss, less surgical site infection, shorter hospital stays, and higher negative resection margins than open resection. Robotic surgery also has lower conversion rates, lower blood loss, lower rates of reoperation, and higher negative circumferential margins than laparoscopic surgery. Robotic surgery had longer operation times and higher costs than open and laparoscopic surgery. There were no differences in other complications, mortality, and survival between robotic surgery and the open or laparoscopic approach. However, heterogeneity between studies was moderate to high in some analyses. The robotic approach can be the method of choice for centers planning to change from open to minimally invasive rectal surgery. The higher costs of robotic surgery should be considered as a substitute for laparoscopic surgery (PROSPERO: CRD42022381468).

## 1. Introduction

Colorectal cancer is the third most common cancer worldwide, and up to 35% of colorectal cancer cases are rectal carcinomas [1,2]. In 2020, 700,000 cases of rectal cancer and approximately 340,000 related deaths were reported worldwide [2]. Due to this high prevalence, the diagnosis and treatment of rectal cancer have received much attention. Surgical resection, including sphincter-preserving proctectomy with total mesorectal excision (TME) or abdominoperineal excision of the rectum, remain the mainstay treatments [3]. Treatment outcomes can also be improved by adjuvant or neo-adjuvant chemoradiotherapy according to the tumor extension and stage of the disease. [4]. Open and laparoscopic approaches have been used for many years to perform rectal resection in rectal cancers, but robot-assisted surgery has been developed to improve minimally invasive treatment [5,6,7].

Several studies have demonstrated that laparoscopic and open rectal surgery have comparable perioperative morbidity and mortality, but that the laparoscopic approach has lower intraoperative blood loss, faster recovery and shorter hospital stays than the open approach [8,9,10]. However, despite these advantages, surgeons need more training to perform laparoscopic rectal surgeries [11] and laparoscopy has technical limitations, such as reduced manual dexterity, two-dimensional view, and restricted flexibility of the laparoscopic arms in the narrow pelvic space, which impairs the resection margin [12,13,14]. These limitations were overcome by the introduction of robotic surgery for rectal resection in 2001 [14], which provided better dexterity, three-dimensional view of the surgical site, and articulating arms with increased maneuverability [15,16,17]. Moreover, robot-assisted surgery has proven to be an efficient and effective option for treating other pelvic cancers. It offers surgeons better control and increases operative accuracy while reducing physical burden [15,16,17].

Several studies have compared different rectal surgery techniques [6,18,19,20]. As the use of robots in surgical procedures expands, surgical centers are shifting from open or laparoscopic approaches to robot-assisted surgeries in the treatment of rectal cancer. In order to decide which approach is best, surgeons need to know how surgical, pathological, oncological, and cost outcomes differ between robot-assisted surgery and laparoscopic or open surgery. Some systematic reviews and meta-analyses have compared these methods [19,21,22,23,24,25,26,27,28], but a comprehensive review of the outcomes, especially after the learning curve era of the robot-assisted surgery, is still lacking.

The present meta-analysis of randomized controlled trials (RCT) and prospective studies was designed to evaluate the perioperative surgical outcomes, the long-term oncological outcomes, and the costs of robot-assisted surgery with those of open and laparoscopic surgery in patients with rectal cancer.

## 2. Materials and Methods

The present meta-analysis was performed according to recommendations of the Study Center of the German Society of Surgery and Preferred Reporting Items for Systematic Reviews (PRISMA) guidelines [29,30]. The review protocol has been registered in the international prospective register of systematic reviews (PROSPERO: CRD42022381468).

### 2.1. Literature Search

A systematic search in Medline (via PubMed), Web of Science, and CENTRAL databases was performed to identify relevant articles published between inception and October 2022. The reference lists of identified studies were also searched for other relevant articles. No publication time restrictions were applied. The following search terms were used:

(Rectal Cancer* OR Rectum Cancer* OR Rectal Tumor* OR Rectum Tumor* OR Rectum Neoplasm* OR Rectal Neoplasm* OR Total mesorectal exicision OR TME OR rectum OR mesorectal OR Rectal Neoplasms [mesh] OR Rectum/surgery [mesh]) AND (laparoscop* OR minimal access surgery OR minimally access OR minimally invasive OR Laparoscopy [mesh] OR Minimally Invasive Surgical Procedures [mesh]) AND (Robotic OR robotic surgery OR robotic-assisted surgery OR robot-assisted surgery OR robotic-assisted OR robotassisted OR robot assisted OR da vinci OR davinci OR Robotic Surgical Procedures [mesh] OR Surgical Procedures, Operative [mesh] OR Minimally Invasive Surgical Procedures [mesh]).

### 2.2. Eligibility Criteria

The eligibility criteria were formulated according to the PICOS strategy:

Population: adult patients with rectal neoplasia undergoing elective rectal resection.

Intervention: robot-assisted rectal resection.

Comparators: open rectal resection in meta-analysis A and laparoscopic rectal resection in meta-analysis B.

Outcome: intraoperative outcomes (including intraoperative complications, conversion to open surgery, estimated blood loss, and operation time) and postoperative surgical outcomes (including histopathological outcomes, postoperative pain, postoperative complications, reoperation, length of hospital stay, and up to 90-day mortality), survival, and cost outcomes.

Study design: RCT and prospective studies comparing the techniques.

Studies that did not report data suitable for meta-analysis were excluded. Retrospective studies, review articles, studies using cadavers or animals, and studies that reported similar data on the same patient population were all excluded. No language restriction was applied to included studies.

### 2.3. Study Selection and Data Extraction

All titles and abstracts were screened for inclusion according to the abovementioned criteria by two independent investigators (ADM, RN). Then, the full texts of selected articles were screened for in-depth review by two investigators (EA, ADM) and data were extracted from eligible articles into a pre-structured Microsoft Excel data sheet (Version 2019, Microsoft). Disagreements were resolved by consultation with the first author (EK).

### 2.4. Definition of Extracted Data

**Characteristics:** We extracted data on the year of publication, first author’s name or study name, country of origin, study design, methods of rectal resection (robotic, open, or laparoscopic), age, gender, American Society of Anesthesiologists (ASA) score, body mass index (BMI, kg/m^2^), sphincter preservation, and follow-up duration from all included studies.

**Intraoperative outcomes:** Intraoperative complications, conversion rate to open surgery, estimated blood loss, and operation time.

**Postoperative outcomes:** Postoperative overall and major complications (Clavien–Dindo score ≥ III), including respiratory complications, urinary complications, ileus, anastomotic leakage, surgical site infection, postoperative pain (based on visual analog scale score), reoperation, and length of hospital stay.

**Histological outcomes:** Completeness of TME, rate of microscopic margin-free resection (R0), rate of negative circumferential resection margin (CRM), proximal resection margin, distal resection margin, and number of harvested lymph nodes.

**Mortality and survival outcomes:** Up to 90-day mortality; 1- and 3-year recurrence-free survival (RFS); and 1- and 3-year overall survival (OS).

**Operative costs:** Overall costs of surgery.

### 2.5. Quality Assessment

The quality of the studies was evaluated using the Cochrane risk-of-bias (RoB2) tool [31] for RCTs and the Risk of Bias in Non-randomized Studies of Interventions (ROBINS-I) tool for non-randomized studies [32]. The he overall quality of the evidence of the results was assessed using the Grades of Recommendation, Assessment, Development, and Evaluation (GRADE) system [33]. The quality assessment was performed by 2 independent reviewers (EA and RN) and disagreements were resolved by consultation with the first author (EK).

### 2.6. Statistical Analysis

Data analysis was performed using RevMan version 5.3 (Nordic Cochrane Centre, Cochrane Collaboration, Copenhagen, Denmark). Two different meta-analyses were performed: Meta-analysis A (robotic vs. open rectal resection) and meta-analysis B (robotic vs. laparoscopic rectal resection). Dichotomous data were presented as odds ratios (ORs) and continuous data as mean differences (MDs). A random-effects model was used to control the heterogeneity of included studies. With regards to dispersion of effect sizes, an I^2^ index > 75% demonstrated high amount of heterogeneity, and 50% < I^2^ ≤ 75%, 25% < I^2^ ≤ 50%, and I^2^ ≤ 25% showed moderate, low, and no clinically relevant heterogeneity among studies, respectively. A *p*-value of <0.05 was considered statistically significant in all analyses.

## 3. Results

### 3.1. Literature Search and Study Characteristics

**Figure 1:**
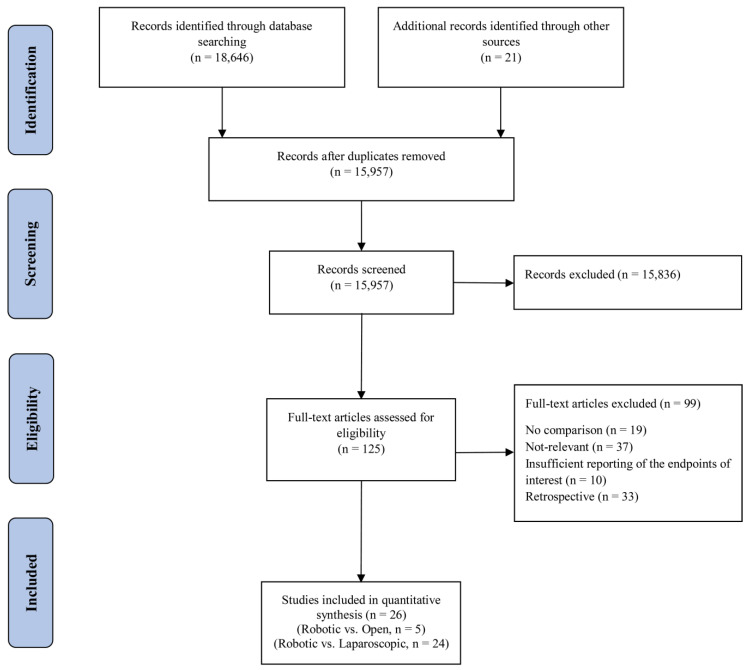
PRISMA flow chart showing the selection of articles for review.

The systematic search retrieved 18,667 articles. After removing duplicates, the titles and abstracts of 15,957 articles were screened for further review, 125 of which were selected for in-depth review. After this, 15 RCTs [6,17,19,20,23,34,35,36,37,38,39,40,41,42,43] and 11 prospective studies [18,44,45,46,47,48,49,50,51,52,53] complied with our eligibility criteria and were included in the final qualitative and quantitative meta-analysis. This selection process is depicted in the PRISMA flowchart (Figure 1). The characteristics of the included studies are shown in Table 1. Overall, 6922 patients were included in this meta-analysis, 5348 of which compared laparoscopic and robot-assisted surgeries and 1574 of which compared open and robot-assisted surgeries. The included studies were conducted in 13 countries. After review, we found that Jayne et al. [19] and Corrigan et al. [34] used the same data from the ROLARR randomized clinical trial, so we analyzed both articles as the ROLARR randomized clinical trial and extracted the outcomes from the cumulative data.

### 3.2. Risk of Bias Assessment

According to the RoB2 tool, 11 of 15 RCTs explained the method of random sequence generation sufficiently. These methods included computer-generated random numbers, minimization, and sealed envelopes. These 11 studies were considered to have a low risk of bias. Three studies were evaluated as having some concerns of bias. Random allocation concealment was reported in detail in five studies and the risk of bias was evaluated as low in these studies. Eight studies showed some concerns of bias and two studies were considered as having a high risk of bias. Eight studies did not report enough on the blinding methods of participants, personnel, or outcome assessment, so were considered to have some concerns of bias. Seven studies were considered to have a high risk of bias in blinding of participants and personnel, and three studies in blinding of outcome assessment. Five studies reported adequate methods for blinding the outcome assessor, which decreased the higher risk of bias due to insufficient blinding of participants and personnel. These studies were judged as having a low risk of bias. Eight RCTs had no missing data or adequately explained why the missing data would not affect the outcome; these studies were considered as having a low risk of bias. The seven remaining RCTs didn’t provide enough data to evaluate the incompleteness of data and had some concerns for bias. Eight RCTs presented a previously published study protocol or trial registration and were considered as having a low risk of bias due to selective reporting. The other seven RCTs didn’t report enough data to judge the risk of reporting bias, and were considered as having some concerns of bias. Regarding other sources of bias, seven studies reported adequate data about funding, industrial bias, sample size calculation, and conflict of interests, so were considered as having a low risk of bias. One study showed a high risk of bias and seven studies did not report sufficient data and were judged as having some concerns of bias. Overall, seven studies were judged as having a high risk of bias (46.7%) and the remaining eight studies as having some concerns of bias (53.3%) (Table 2).

The quality assessment of the 11 non-randomized prospective studies is presented in Table 3. In the “confounding” domain, the risk of bias was moderate in nine studies (82%), and serious in two studies (18%). In the “selection of participants” domain, the risk of bias was low in two studies (18%) and moderate in nine studies (82%). In the “classification of interventions”, “deviations from intended interventions”, and “bias in outcome measurements” domains, the risk of bias was low in seven studies (63.6%) and moderate in four studies (36.4%). In the “bias due to missing data” domain, the risk of bias was moderate in nine studies (82%) and serious in two studies (18%). In the “selection of the reported results” domain, the risk of bias was low in two studies, moderate in eight studies (73%), and serious in one study (9%). The overall risk of bias was moderate in seven studies (63.6%) and serious in four studies (36.4%). The GRADE assessments are presented in Supplementary Tables S1 and S2. The quality of evidence was judged as low and very low for the majority of analyzed outcomes and were only moderate in two of the evaluated outcomes.

Domain 1: Bias due to confounding; Domain 2: Bias in selection of participants into the study; Domain 3: Bias in classification of interventions; Domain 4: Bias due to deviations from intended interventions; Domain 5: Bias due to missing data; Domain 6: Bias in measurements of outcomes; Domain 7: Bias in selection of the reported result.

### 3.3. Quantitative Analysis

#### 3.3.1. Meta-Analysis A: Robotic vs. Open Rectal Resection

In the final screening, five articles compared the outcomes of robotic and open surgery. These studies involved 1574 patients and the meta-analysis results are described below.

##### Intraoperative Outcomes

**Figure 2:**
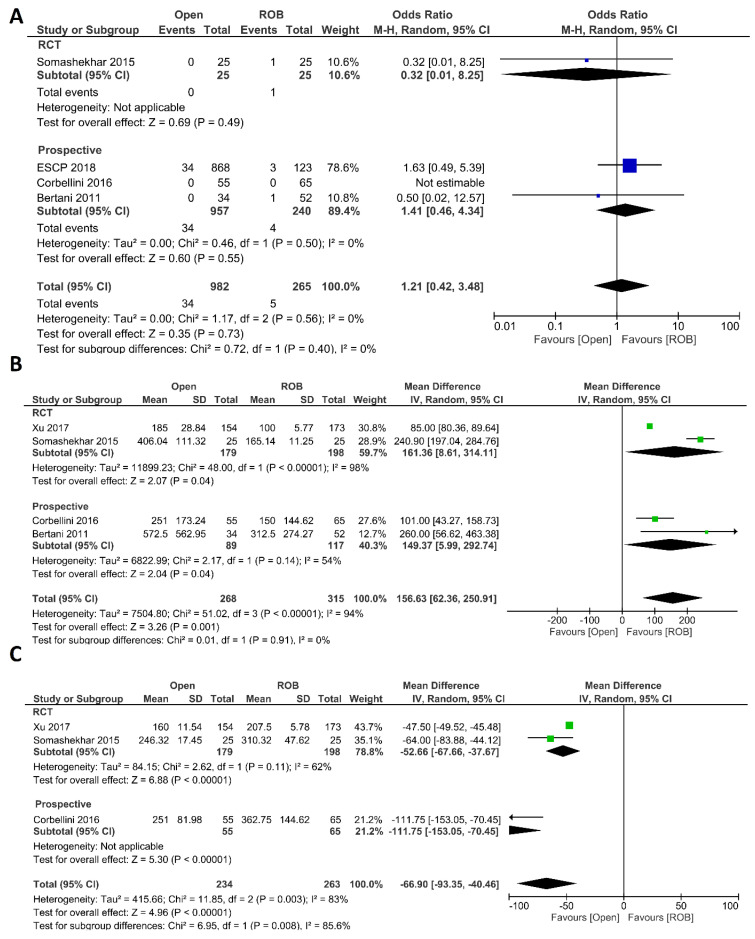
Forest plots comparing (**A**) intraoperative complications [18,20,44,46], (**B**) estimated blood loss [18,20,40,46], and (**C**) operation time [18,20,40] between robotic and open rectal resection (blue boxes representing odd ratios, green boxes representing mean differences, arrows representing 95% confidence intervals, and diamonds representing point estimates of pooled odd ratios or mean differences).

Intraoperative complications, estimated blood loss, and operation time were investigated as intraoperative outcomes. Four studies including 1247 participants reported the rate of intraoperative complications, 982 of whom were in the open surgery group and 265 of whom were in the robotic surgery group. In total, 34 patients (3.46%) had intraoperative complications in the open surgery arm, while five patients (1.88%) had intraoperative complications in the robotic surgery arm. However, this difference was not statistically significant (OR = 1.21; 95% CI: 0.42–3.48; *p* = 0.73). No heterogeneity was revealed between studies in this analysis (I^2^ = 0%; *p* = 0.56) (Figure 2A). Estimated blood loss was reported in 583 participants from four studies. Blood loss was significantly lower in the robotic surgery group than in the open surgery group (MD = 156.63, 95% CI: 62.36–250.91; *p* = 0.001). The same findings were observed in the subgroup analysis of RCTs (*p* < 0.001). Heterogeneity between these studies was high (I^2^ = 94%, *p* < 0.001) (Figure 2B). Three studies reported the operating time, and a pooled analysis revealed a longer operation time in the robotic surgery group than in the open surgery group (MD = −66.90, 95% CI: −93.35–−40.46; *p* < 0.00001) with high heterogeneity (I^2^ = 83%, *p* = 0.003) (Figure 2C).

##### Postoperative Outcomes

**Figure 3:**
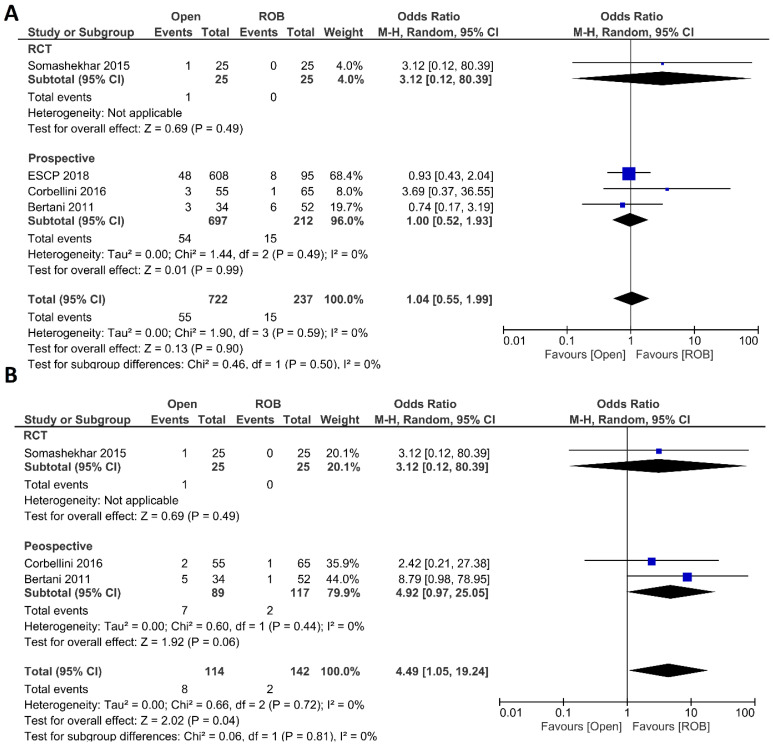
Forest plots comparing (**A**) anastomotic leakage [18,20,44,46] and (**B**) surgical site infection [18,20,46] between robotic and open rectal resection (blue boxes representing odd ratios, arrows representing 95% confidence intervals, and diamonds representing point estimates of pooled odd ratios or mean differences).

**Figure 4:**
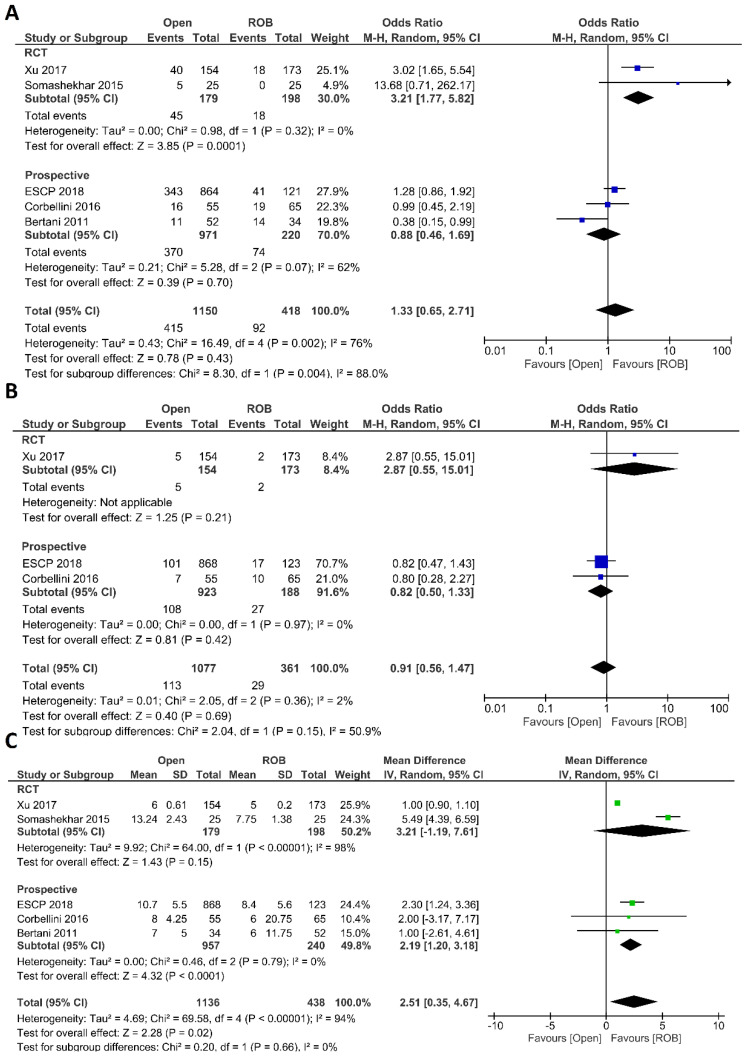
Forest plots comparing (**A**) postoperative complications [18,20,40,44,46], (**B**) major complications [18,40,44], and (**C**) length of hospital stay [18,20,40,44,46] between robotic and open rectal resection (blue boxes representing odd ratios, green boxes representing mean differences, arrows representing 95% confidence intervals, and diamonds representing point estimates of pooled odd ratios or mean differences).

Anastomotic leakage, surgical site infection, postoperative complications, major complications, and length of hospital stay were evaluated as postoperative outcomes. Four included studies reported anastomotic leakage, and 722 patients in the open surgery arm and 237 patients in the robotic surgery arm were entered in the meta-analysis. There were no significant differences in anastomotic leakage between the open surgery and robotic surgery group (OR = 1.04; 95 % CI: 0.55–1.99; *p* = 0.9), and no evidence of heterogeneity was found among the included studies (I^2^ = 0%; *p* = 0.59) (Figure 3A). Three studies including 256 patients reported surgical site infection and there was a significantly higher surgical site infection in individuals who underwent open surgery than those who underwent robotic surgery (7.1% vs. 1.4%; OR = 4.49; 95 % CI: 1.05–19.24; *p* = 0.04). While not statistically significant, the difference was also observed in the sub-analysis of non-randomized prospective studies (*p* = 0.06). No heterogeneity was found among the studies (I^2^ = 0%; *p* = 0.72) (Figure 3B). Five studies reported postoperative complications in a total of 1568 participants. Postoperative complications were reported in 38% of patients who underwent open surgery and in 22% of patients who underwent robotic surgery. However, this difference was not statistically significant (OR = 1.33; 95% CI: 0.65–2.71; *p* = 0.43). Subgroup analysis of two RCTs revealed a significant difference with postoperative complications in 25% of patients in the open surgery group and in 10% of patients in the robotic surgery group (OR = 3.21; 95% CI: 1.77–5.82; *p* < 0.001). The analysis revealed high heterogeneity between the studies in this analysis (I^2^ = 76%; *p* = 0.002) (Figure 4A). Major complications were reported in three studies, including 1077 patients in the open surgery arm and 361 patients in the robotic surgery arm. The rate of major complications was 8.0% in the robotic surgery arm and 10.5% in the open surgery arm; this difference was not significant and no heterogeneity was detected between the studies (OR = 0.91; 95% CI: 0.56–1.47; *p* = 0.69; I^2^ = 2%, *p* = 0.36) (Figure 4B). The duration of hospitalization was reported in five studies—two RCTs and three prospective studies—comprising 1574 patients. The hospital stay was significantly shorter in the robotic surgery group than the open surgery group in the final analysis of the pooled data, which indicated a 2.5-day shorter hospital stay in the robotic surgery group (MD = 2.56; 95% CI: 0.31–4.81; *p* = 0.03). The meta-analysis showed high heterogeneity between studies (I^2^ = 97%; *p* < 0.001) (Figure 4C).

##### Histological Outcomes

**Figure 5:**
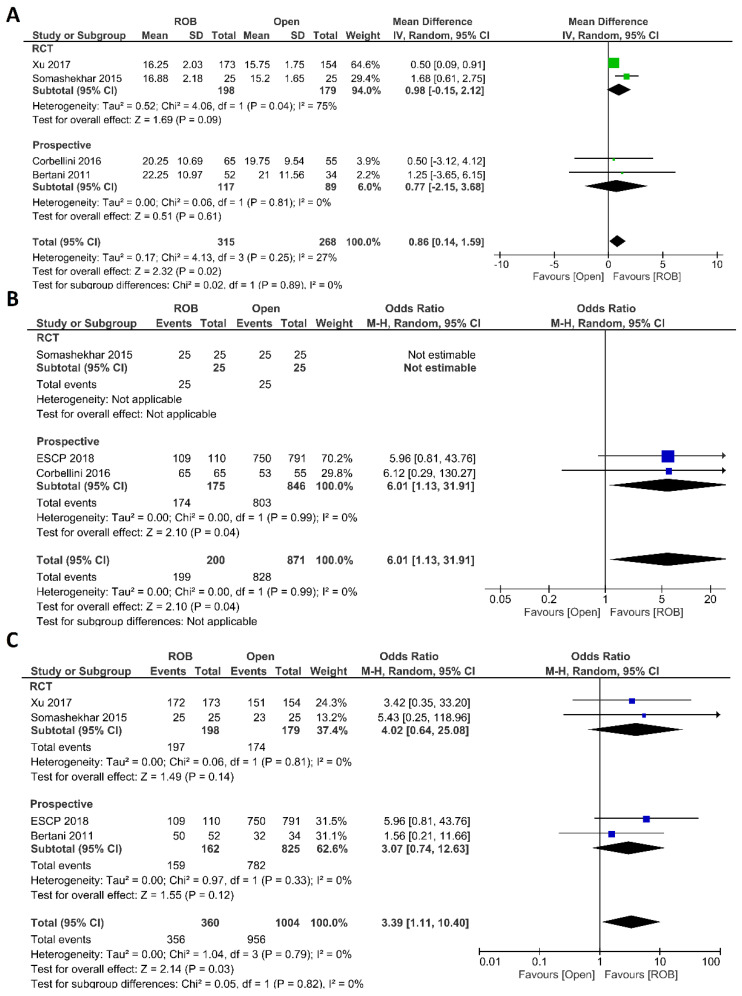
Forest plots comparing (**A**) number of harvested lymph nodes [18,20,40,46], (**B**) microscopic margin-free (R0) resection [18,20,44] and (**C**) circumferential resection margin (CRM) [20,40,44,46] between robotic and open rectal resection (blue boxes representing odd ratios, green boxes representing mean differences, arrows representing 95% confidence intervals, and diamonds representing point estimates of pooled odd ratios or mean differences).

Regarding histological outcomes, we assessed the number of harvested lymph nodes, the rate of R0 resection and CRM negative resection. Four studies comprising 583 patients reported the number of harvested lymph nodes; 315 of these patients were in the robotic surgery group and 268 were in the open surgery group. The meta-analysis showed significantly more harvested lymph nodes in the robotic surgery group than in the open surgery group (MD = 0.86; 95% CI: 0.14–1.59; *p* = 0.02). Low heterogeneity was detected between studies in this analysis (I^2^ = 27%, *p* = 0.25) (Figure 5A). Three studies reported R0 resection rates in 1071 participants, 871 of whom underwent open surgery and 200 of whom underwent robotic surgery. Approximately six times more negative margins were achieved in the robotic surgery group than in the open surgery group (OR = 6.01; 95% CI: 1.13–31.91; *p* = 0.04). No heterogeneity between the studies was detected (I^2^ = 0%; *p* = 0.99) (Figure 5B). The rate of negative CRM was evaluated in four studies comprising 360 patients in the robotic surgery group and 1004 patients in the open surgery group. The meta-analysis showed that the rate of negative CRM was three times higher in patients who underwent robotic surgery than in those who underwent open surgery (OR = 3.39; 95% CI: 1.11–12.26; *p* = 0.03). No heterogeneity was detected between studies in this analysis (I^2^ = 0%; *p* = 0.79) (Figure 5C).

##### Mortality and Survival Outcomes

Five studies reported perioperative mortality [18,20,40,44,46]; no death was mentioned in either surgical group, so no pooled analysis was performed. RFS and OS were reported in one study by Corbellini et al. This study comprised 65 patients in the robotic surgery group and 55 patients in the open surgery group. The one-year RFS was 97% in the open surgery group and 95% in the robotic surgery group and the three-year RFS was 84% in the open surgery group and 80% in the robotic surgery group. The difference was not significant. In the open surgery group, the one-year OS was 98%, which decreased to 93% after three years. In the robotic surgery group, the one-year OS was similar to that in the open surgery group but decreased to 87% after three years.

##### Operative Costs

Operative costs were compared between robotic and open surgery groups in one study by Bertani et al. This study performed cost analysis in 86 rectal cancer patients, and the mean operative cost of robotic surgery was 12,680 USD, which was 1530 USD higher than that of open surgery.

#### 3.3.2. Meta-Analysis B: Robotic vs. Laparoscopic Rectal Resection

Twenty-three articles compared robotic and laparoscopic surgery in a total of 5348 patients.

##### Intraoperative Outcomes

**Figure 6:**
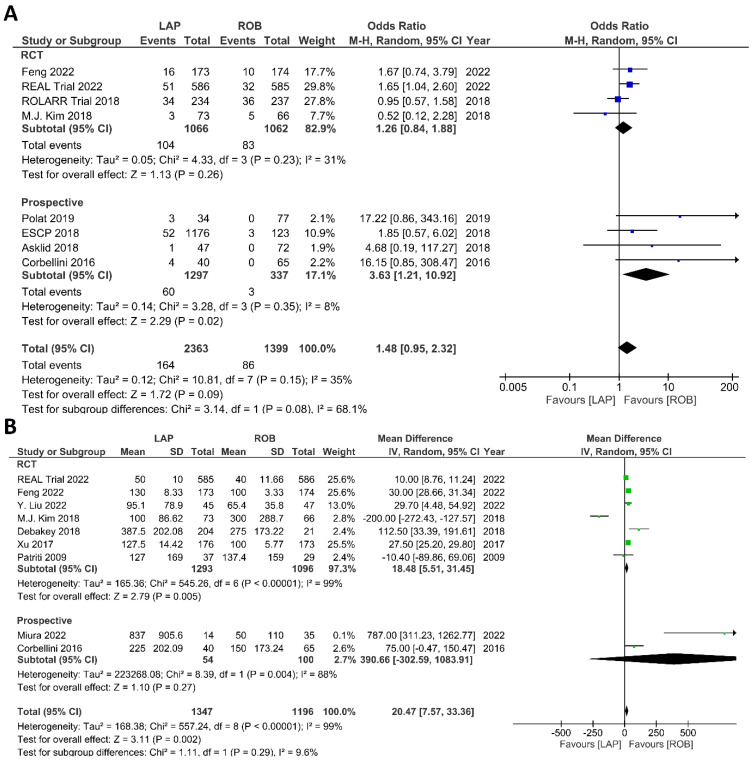
Forest plots comparing (**A**) intraoperative complications [17,18,19,34,41,42,44,45,52] and (**B**) estimated blood loss [17,18,35,37,40,41,42,43,51] between robotic and laparoscopic rectal resection (blue boxes representing odd ratios, green boxes representing mean differences, arrows representing 95% confidence intervals, and diamonds representing point estimates of pooled odd ratios or mean differences).

**Figure 7:**
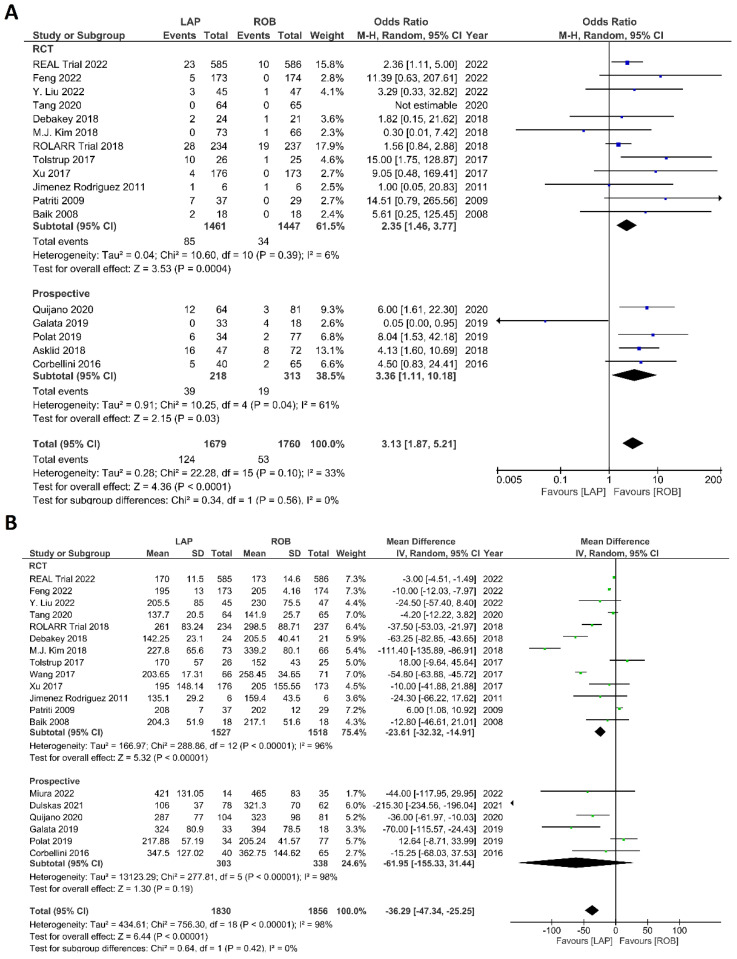
Forest plots comparing (**A**) conversion to open surgery [6,17,18,19,23,34,35,36,37,38,40,41,42,43,45,48,52,53] and (**B**) operation time [6,17,18,19,23,34,35,36,37,38,39,40,41,42,43,47,48,51,52,53] between robotic and laparoscopic rectal resection (blue boxes representing odd ratios, green boxes representing mean differences, arrows representing 95% confidence intervals, and diamonds representing point estimates of pooled odd ratios or mean differences).

Intraoperative outcomes comprised intraoperative complications, conversion rate to open surgery, estimated blood loss during surgery, and operation time. Eight studies reported intraoperative complications in 3762 patients, and the meta-analysis did not reveal significant differences between the robotic surgery and laparoscopic surgery groups (OR = 1.48; 95% CI: 0.95–2.32; *p* = 0.26). The estimated heterogeneity between studies was low in this analysis (I^2^ = 35%; *p* = 0.09) (Figure 6A). Estimated blood loss was reported in nine studies, comprising 2543 patients. The estimated blood loss was significantly less in robotic surgery than in laparoscopic surgery (MD = 20.47; 95% CI: 7.57–33.36; *p* = 0.002). However, high heterogeneity was detected between studies in this analysis (I^2^ = 99%; *p* < 0.001) (Figure 6B). The conversion to open surgery was reported in 17 studies, comprising 1760 patients in the robotic surgery group and 1679 patients in the laparoscopic surgery group. Conversion to open surgery was reported in 53/1760 (3.01%) patients in the robotic surgery group and in 124/1679 (7.38%) patients in the laparoscopic surgery group. The meta-analysis showed that conversion to open surgery was three-times more common during laparoscopic surgery than during robotic surgery (OR = 3.13; 95% CI: 1.87–5.21; *p* < 0.0001). Low heterogeneity was detected between studies in this analysis (I^2^ = 33%; *p* = 0.10) (Figure 7A). Operation duration was reported in 19 studies comprising 3686 patients, 1856 of whom underwent robotic surgery and 1830 of whom underwent laparoscopic surgery. The operation duration was significantly longer in the robotic surgery group (MD = −36.29; 95% CI: −47.34 to −25.25; *p* < 0.001). This difference was also detected in the subgroup analysis of RCTs. High heterogeneity was detected between studies in this analysis (I^2^ = 98%; *p* < 0.001) (Figure 7B).

##### Postoperative Outcomes

**Figure 8:**
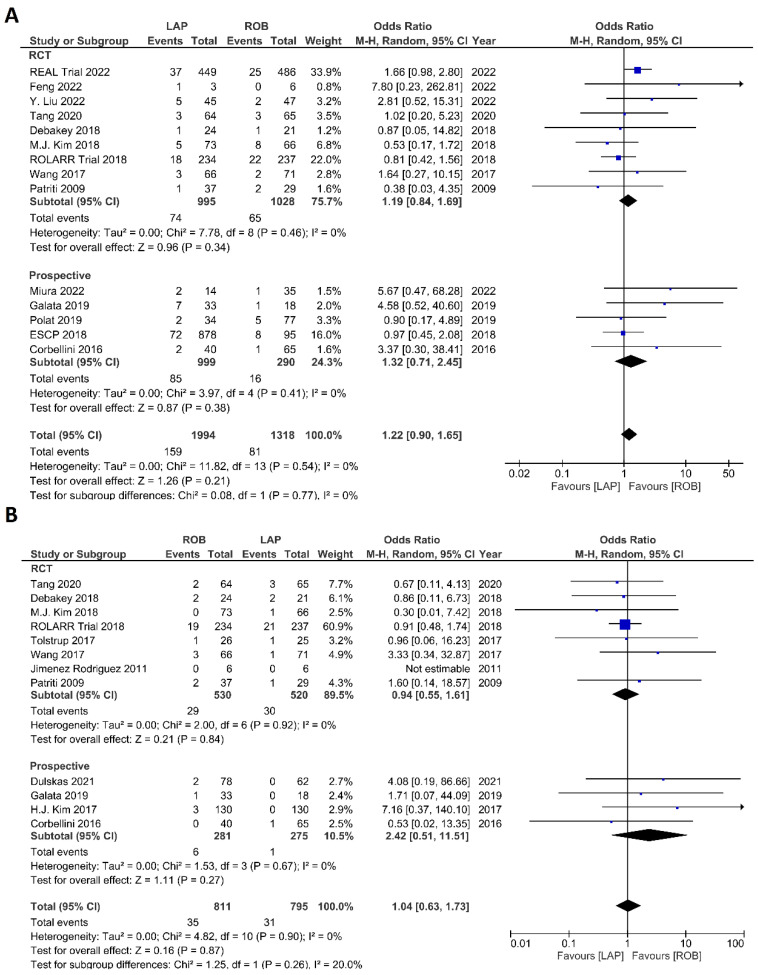
Forest plots comparing (**A**) anastomotic leakage [17,18,19,23,34,35,37,39,41,42,43,44,48,51,52] and (**B**) surgical site complications [54] between robotic and laparoscopic rectal resection (blue boxes representing odd ratios, arrows representing 95% confidence intervals, and diamonds representing point estimates of pooled odd ratios or mean differences).

**Figure 9:**
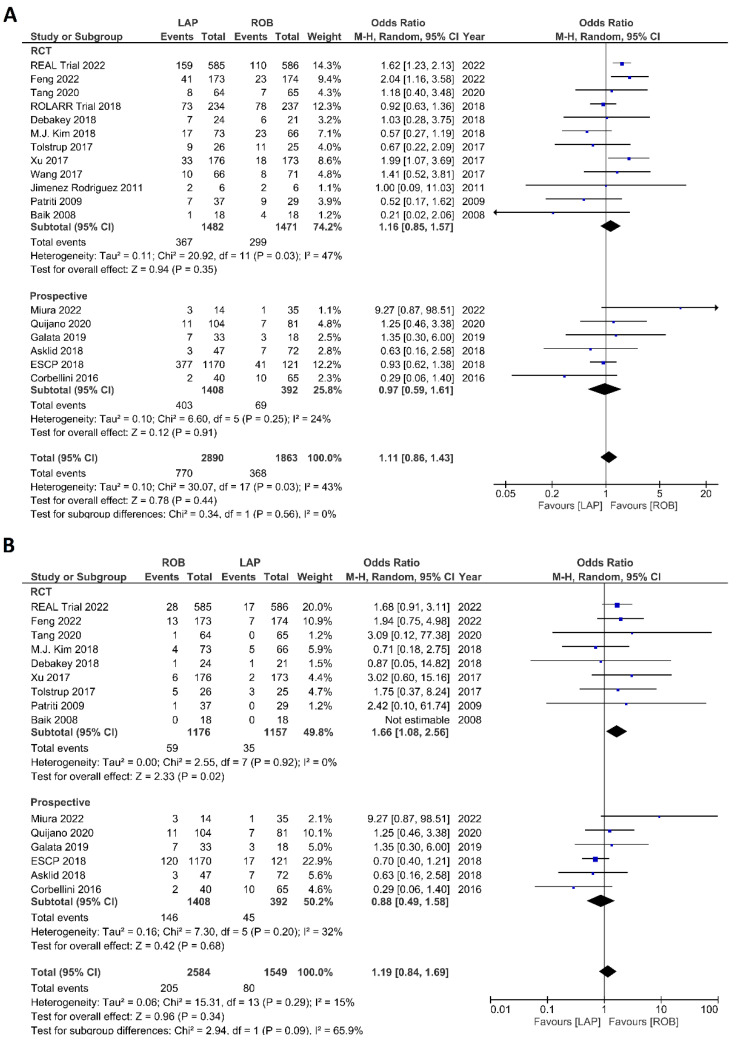
Forest plots comparing (**A**) postoperative complications [6,17,18,19,23,34,35,36,37,38,39,40,41,42,44,45,48,51,53] and (**B**) major complications [6,17,18,23,35,37,38,40,41,42,44,45,48,51,53] between robotic and laparoscopic rectal resection (blue boxes representing odd ratios, arrows representing 95% confidence intervals, and diamonds representing point estimates of pooled odd ratios or mean differences).

**Figure 10:**
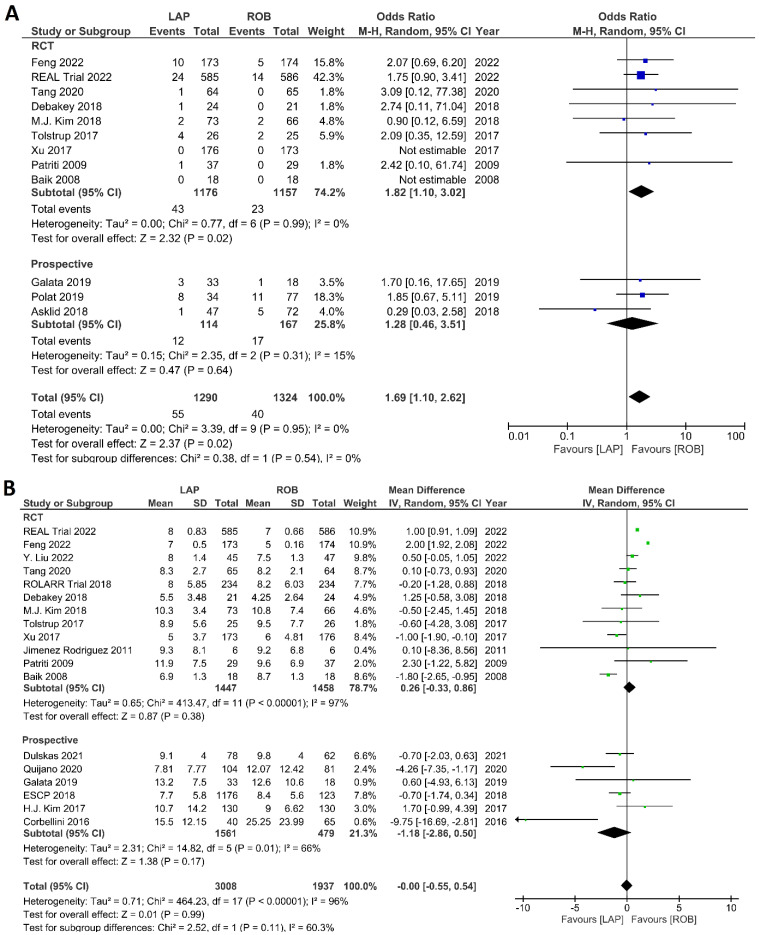
Forest plots comparing (**A**) reoperation [6,17,23,35,37,38,40,41,42,45,48,52] and (**B**) length of hospital stay [6,17,18,19,23,34,35,36,37,38,40,41,42,43,44,47,48,49,53] between robotic and laparoscopic rectal resection (blue boxes representing odd ratios, green boxes representing mean differences, arrows representing 95% confidence intervals, and diamonds representing point estimates of pooled odd ratios or mean differences).

The investigated postoperative outcomes were anastomotic leakage, surgical site infection, postoperative complications, major complications, reoperation rate, and length of hospital stay. Fourteen studies reported on anastomotic leakage, which was detected in 81 of 1318 patients (6.14%) undergoing robotic surgery and in 159 of 1994 patients (7.97%) undergoing laparoscopic surgery. The difference in anastomotic leakage was not significant between the two arms (OR = 1.22; 95% CI: 0.90–1.65; *p* = 0.21). Heterogeneity was not detected between studies in this analysis (I^2^ = 0%; *p* = 0.54) (Figure 8A). Surgical site infection was reported in 12 studies. The pooled analysis revealed surgical site infection in 31/795 (3.9%) patients undergoing robotic surgery and in 35/811 (4.3%) patients undergoing laparoscopic surgery. This difference was not significant (OR = 1.04; 95% CI: 0.63–1.73; *p* = 0.87) and no heterogeneity was detected between studies in this analysis (I^2^ = 0%; *p* = 0.90) (Figure 8B). Postoperative complications were reported in 18 studies, with 1865 patients in the robotic surgery group and 2890 patients in the laparoscopic surgery group. The pooled analysis revealed no significant differences in postoperative complications between the robotic surgery group and the laparoscopic surgery group (OR = 1.11; 95% CI: 0.86–1.43; *p* = 0.44). Low heterogeneity was detected between the studies in this analysis (I^2^ = 43%; *p* = 0.03) (Figure 9A). Major complications were reported in 15 studies comprising 4133 patients. The meta-analysis showed no significant difference in major complications between the robotic surgery group and the laparoscopic surgery group (OR = 1.19; 95% CI: 0.84–1.69; *p* = 0.34). Low heterogeneity was detected between the studies in this analysis (I^2^ = 15%; *p* = 0.29). However, the subgroup analysis of RCTs revealed a significantly higher rate of major complications in laparoscopic surgery group (OR = 1.66; 95% CI: 1.08–2.56; *p* = 0.02; I^2^ = 0%; *p* = 0.92) (Figure 9B). Reoperation rate was reported in 12 studies comprising 2614 patients, 1324 of whom underwent robotic surgery and 1290 of whom underwent laparoscopic surgery. The meta-analysis showed that 1.5-times more reoperations were performed in the laparoscopic surgery group than in the robotic surgery group (OR = 1.69; 95% CI: 1.10–2.62, *p* = 0.02). There was no evidence of heterogeneity between the studies in this analysis (I^2^ = 0%; *p* = 0.95) (Figure 10A). The length of hospital stay was reported in 18 studies. A pooled analysis of 4945 patients demonstrated no significant difference in the length of hospital stay between the robotic surgery group and the laparoscopic surgery group (MD = −0.00; 95% CI: −0.55–0.54; *p* = 0.99). Heterogeneity was high between the analyzed studies (I^2^ = 96%; *p* < 0.001) (Figure 10B).

##### Histological Outcomes

**Figure 11:**
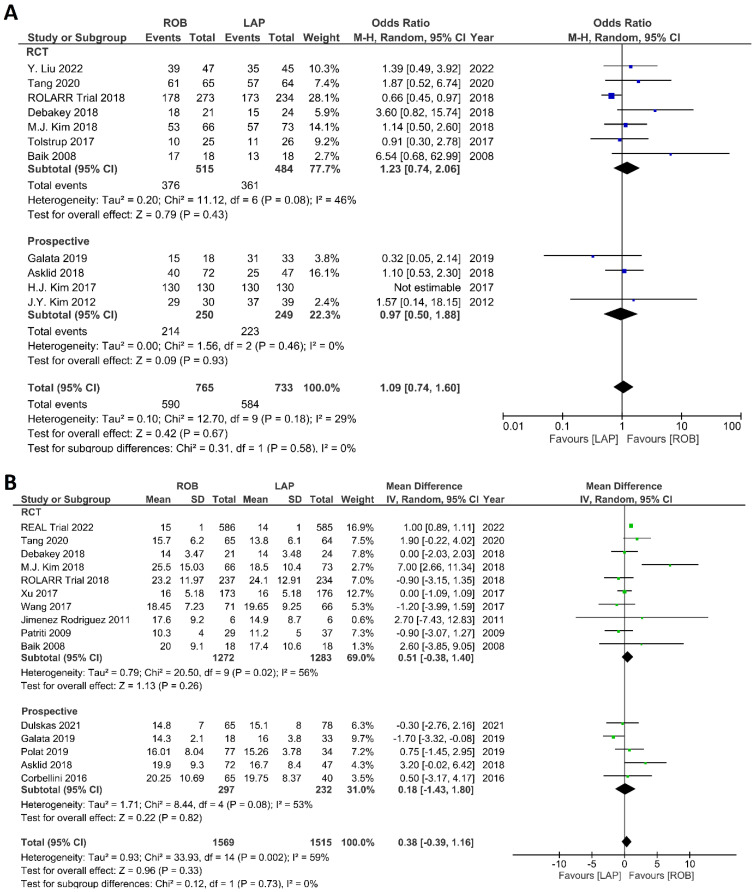
Forest plots comparing (**A**) TME completeness [6,17,19,23,34,35,38,43,45,48,49,50] and (**B**) total harvested lymph nodes [6,17,18,19,23,34,35,36,37,39,40,41,45,47,48,52] between robotic and laparoscopic rectal resection (blue boxes representing odd ratios, green boxes representing mean differences, arrows representing 95% confidence intervals, and diamonds representing point estimates of pooled odd ratios or mean differences).

**Figure 12:**
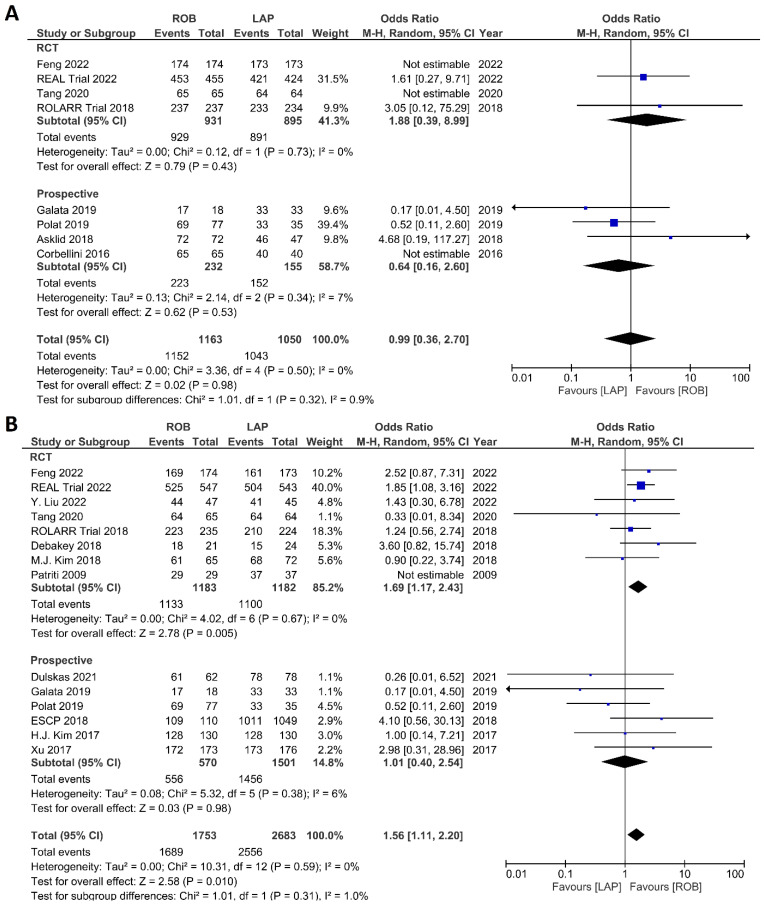
Forest plots comparing (**A**) microscopic margin-free resection (R0 resection) [18,19,23,34,41,42,45,48,52] and (**B**) circumferential resection margin (CRM) [17,19,23,34,35,37,40,41,42,43,44,47,48,49,52] between robotic and laparoscopic rectal resection (blue boxes representing odd ratios, arrows representing 95% confidence intervals, and diamonds representing point estimates of pooled odd ratios or mean differences).

The investigated histological outcomes were completeness of TME, rate of R0 resection, rate of CRM negative resection, number of harvested lymph nodes, and proximal and distal resection margins. The completeness of TME was reported in 11 studies comprising 1498 patients. The pooled analysis revealed similar rates of complete TME in the robotic surgery and laparoscopic surgery groups (OR = 1.09; 95% CI: 0.74–1.60; *p* = 0.67). Low heterogeneity was found between the studies in this analysis (I^2^ = 29%; *p* = 0.18) (Figure 11A). The number of excised lymph nodes was reported in 15 studies comprising 3084 patients (1569 patients in the robotic surgery group and 1515 patients in the laparoscopic surgery group). The pooled analysis showed no significant difference in the number of excised lymph nodes between the two groups (MD = 0.38; 95% CI: −0.39–1.16; *p* = 0.33). Moderate heterogeneity was detected between the studies in this analysis (I^2^ = 59%; *p* = 0.002) (Figure 11B). The rate of R0 resection was reported in 8 studies comprising 2213 participants. The meta-analysis showed no significant difference in R0 resection between the robotic and laparoscopic arms (OR = 0.99; 95% CI: 0.36–2.70; *p* = 0.98). No heterogeneity was detected between the studies in this analysis (I^2^ = 0%; *p* = 0.50) (Figure 12A). The rate of CRM negative resection was reported in 14 studies including 4436 patients (1753 patients in the robotic surgery group and 2683 patients in the laparoscopic surgery group). The pooled analysis showed significantly 1.5-times more negative CRM resection in the robotic surgery group than in the laparoscopic surgery group (OR = 1.56; 95% CI: 1.11–2.20; *p* = 0.01). No heterogeneity was detected between the studies in this analysis (I^2^ = 0%; *p* = 0.59) (Figure 12B). Furthermore, no significant differences were detected in proximal and distal resection margins between robotic and laparoscopic rectal resection. Results of these meta-analyses are presented in the Supplementary Material.

##### Mortality and Survival Outcomes

**Figure 13:**
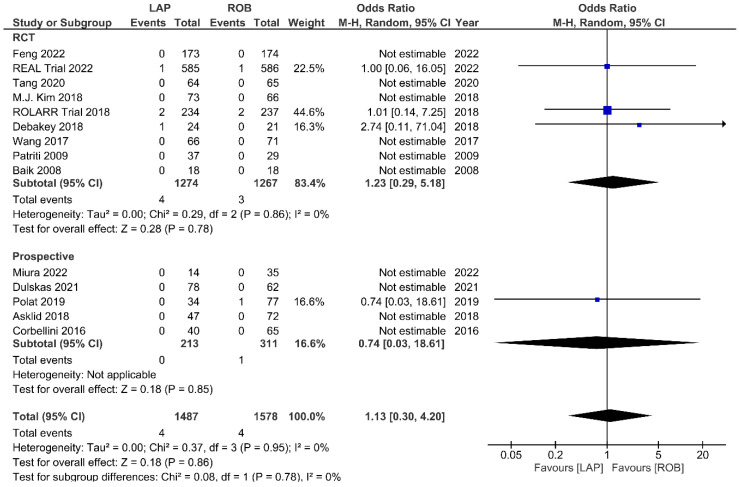
Forest plots comparing up to 90-day mortality [6,17,18,19,23,34,35,37,39,41,42,45,47,51,52] between robotic and laparoscopic rectal resection (blue boxes representing odd ratios, arrows representing 95% confidence intervals, and diamonds representing point estimates of pooled odd ratios or mean differences).

Fourteen studies reported the perioperative mortality within 30 days or 90 days after surgery. Ten of these studies reported no deaths in both groups, and the meta-analysis confirmed no significant differences in perioperative mortality between the groups (OR = 1.13; 95% CI: 0.30–4.20; *p* = 0.86). No heterogeneity was detected between studies in this analysis (I^2^ = 0%; *p* = 0.95) (Figure 13). One-year RFS was evaluated in three studies and the meta-analysis showed no significant difference between robotic and laparoscopic surgery (OR = 0.68; 95% CI: 0.28–1.62; *p* = 0.38). No heterogeneity was detected between the studies (I^2^ = 0%; *p* = 0.82) (Supplementary Figure S1). The pooled analysis showed no significant differences in three-year RFS between the robotic surgery and laparoscopic surgery groups (OR = 1.08; 95% CI: 0.39–2.96; *p* = 0.88). Moderate heterogeneity was detected between the studies in this analysis (I^2^ = 52%; *p* = 0.12) (Supplementary Figure S2). OS rates were reported in three studies, comprising 579 patients. The one-year OS rate was higher in the robotic surgery group than in the laparoscopic surgery group, but this difference was not significant (OR = 2.10; 95% CI: 0.54–8.07; *p* = 0.28) (Supplementary Figure S3). No heterogeneity was detected between studies in this analysis (I^2^ = 0%; *p* = 0.64). There were also no significant differences in three-year OS between the robotic surgery group and the laparoscopic surgery group (OR = 1.13; 95% CI: 0.32–4.01; *p* = 0.85) (Supplementary Figure S4). Heterogeneity was low between the studies in this analysis (I^2^ = 48%; *p* = 0.14).

##### Operative Cost

**Figure 14:**
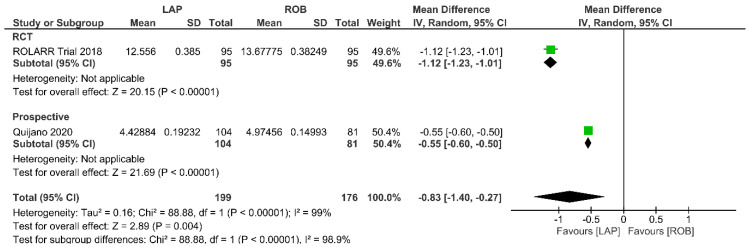
Forest plots comparing operative cost [19,34,53] between robotic and laparoscopic rectal resection (green boxes representing mean differences, arrows representing 95% confidence intervals, and diamonds representing point estimates of pooled odd ratios or mean differences).

Economic variables were only evaluated in the ROLARR RCT and one prospective study. The meta-analysis revealed that the cost of laparoscopic surgery is significantly lower than that of robotic surgery (MD = −0.83; 95% CI: −1.40 to −0.27; *p* = 0.004). High heterogeneity was detected between the studies in this analysis (I^2^ = 99%; *p* < 0.001) (Figure 14).

In meta-analysis B, no significant differences were detected in ileus, pain score, respiratory complications, and urinary complications between the robotic surgery group and laparoscopic surgery group. The results of the pooled analyses are presented in the Supplementary Material.

## 4. Discussion

In recent decades, the increased use of minimally invasive colorectal resection surgeries has improved patient satisfaction, leading to shorter hospital stays, a faster return to normal life, better cosmetic results, and fewer surgical site complications. These benefits have led surgeons to switch from open to minimally invasive techniques when treating these patients [54]. Laparoscopic rectal resection has acceptable surgical outcomes that have improved over time with improvement in instrumentation and increase in surgical experience [8,9,10]. Several studies have shown similar operative and oncological outcomes between laparoscopic surgery and open surgery [8,9,10]; however, some studies have indicated a higher risk of positive CRM and incomplete TME following laparoscopic surgery [12,55,56]. Robotic surgery was introduced to overcome the challenges of laparoscopic resection with advanced technology, 3-D visualization, multiple articulating arms with higher degree of freedom, and enhanced ergonomics. These benefits are particularly relevant to patients with a narrow pelvis, male patients, patients with obesity, and patients with anterior tumors who are treated with neoadjuvant chemotherapy [6,57].

Several RCTs and systematic reviews have shown comparable operative and oncological results of robotic surgery and laparoscopic surgery [19,21,22,23], but there are limitations to this research. While some of these meta-analyses compared robotic, open, and laparoscopic surgery in a pairwise design, others only compared robotic surgery with laparoscopic surgery and included a limited number of studies/participants [21,22,23,24]. Others included retrospective studies [58,59] or evaluated only urological/sexual function and quality of life [58,59] or evaluated only long-term oncological outcomes [22]. Two meta-analyses compared the outcomes of robotic and open resection, but most included studies were retrospective, none were RCTs [27,28] and only the pathological outcomes were evaluated [27]. Two network meta-analyses compared the outcomes of open, laparoscopic, and robotic rectal resection [25,26], but made indirect comparisons between robotic and open approaches. To address these limitations and considering the fast development of robot-assisted colorectal surgery and improvement of its outcomes by increasing the experience of the surgeons, the current meta-analysis was designed to compare directly the surgical, long-term oncological, and economic outcomes of robotic rectal resection with those of open and laparoscopic approaches. Unlike previous research, we included both RCTs and prospective trials, which increased the number of patients included in our meta-analysis (15 RCTs and 11 non-randomized prospective studies) to increase the level of evidence.

Robotic rectal resection showed significantly lower blood loss, reduced surgical site infection, and shorter length of hospital stay, but a longer operation time than open surgery. However, there were no differences in complication rates between the two techniques. Regarding the histological outcomes, robotic surgery had a significantly higher rate of R0 resection and negative CRM resection and a higher number of harvested lymph nodes than open surgery. The operation time was also longer in robotic surgery compared to the laparoscopic procedure, but robotic surgery showed a significantly lower rate of conversion, less intraoperative blood loss, lower rate of reoperation, and higher rate of CRM negative resection. Other outcomes were similar between robotic and laparoscopic surgery.

Conversion to open surgery is associated with higher complication rates, higher mortality rates, and poorer oncological outcomes [60,61,62]. Previous studies have reported similar conversion rates between laparoscopic and robotic surgery [23,26] while others have reported higher conversion rates during laparoscopic surgery than robotic surgery [21,24]. In our study, we found a lower rate of conversion during robotic surgery, which was probably due to better visualization, better operative access to the pelvic area, and better maneuverability and dexterity during surgery [24,63].

We also observed significantly lower estimated blood loss during robotic surgery than during open and laparoscopic surgery. This is probably because the magnified 3-D visualization, instrumental dexterity, and precise resection of the robot improve vascular control during surgery. Higher blood loss during surgery has been associated with a poorer prognosis in colorectal malignancies [64,65], so these findings suggest that robotic surgery may improve the prognosis of patients undergoing rectal resection. Our findings are supported by those of a previous meta-analysis of retrospective studies, which showed lower estimated blood loss with robot-assisted method [26,28]. This previous network meta-analysis also observed lower intraoperative blood loss during robotic surgery than during laparoscopic surgery [26], which is similar to our findings but in contrast to those of some other studies [21,23]. However, minimal differences between groups should be interpreted with caution because it is difficult to assess the exact blood loss in the operation room.

We found that the operative time was longer for robotic surgery than for open and laparoscopic surgery. This may be explained by the time needed to prepare, position, dock, and undock the robotic device before surgery can begin [21,23,26,66]. The operative time in robotic surgery has been shown to decrease with experience [67], and studies have reported that almost 30 operations are needed before the entire surgical team becomes proficient with the robotic device. After this cut-off, the operative time decreases [67]. A recent meta-analysis comparing the effect of the learning curve on the outcomes of robotic and laparoscopic methods in colorectal surgery has shown that the robotic approach has a shorter operative time than the laparoscopic approach when both groups have a similar level of experience [68].

We found a shorter hospital stay and a lower rate of surgical site infection in patients who underwent robotic resection than in patients who underwent open resection. This is likely related to the minimally invasive nature of the robotic method [26,28]. However, we did not observe the any differences in hospital stay and surgical site infections between the robotic and laparoscopic groups. In agreement with our findings, previous reviews have also reported comparable hospitalization time between patients undergoing laparoscopic and robotic surgery [24,25]. However, in contrast to our findings, another meta-analysis reported shorter hospital stays in patients undergoing robotic surgery than in patients undergoing laparoscopic surgery [26]. This heterogeneity might be explained by differences in the level of evidence, included studies, and surgical experience between the two meta-analyses.

We observed better histopathological outcomes following robotic surgery than following open and laparoscopic surgery. For example, the robotic approach had a significantly higher rate of R0 resection and negative CRM resection and a higher number of harvested lymph nodes than the open approach did. Robotic resection had also a higher rate of negative CRM resection than laparoscopic approach. This might be because robots can perform more precise dissection with better visualization, and because pelvic access is improved. However, in contrast to our findings, a previous meta-analysis found no differences in these histopathological outcomes between open and robotic techniques [27]. The superiority of the robotic method shown in our current study may be explained by recent technical and technological improvements of the robotic method and the increased surgical experience with this technique. In spite of the superior histopathological outcomes of robotic procedure, no differences were seen in RFS and OS in the present study. This can be due to the lack of reports on long-term survival outcomes in most of the included studies. Only one study comparing robotic and open procedures and three studies comparing robotic and laparoscopic techniques reported the long term survival outcomes and these studies found also no significant differences in histopathological outcomes between robotic surgery and open or laparoscopic methods. Further studies are needed to evaluate the impact of robotic rectal resection on survival outcomes.

Our meta-analysis showed that operative costs are higher for robotic surgery than for open and laparoscopic surgery, probably due to high costs for the robot and instruments [69]. Creating a more competitive and open market for these surgical devices would decrease the device and maintenance costs [69]. It should also be considered that the cost of robotic devices will decrease with time, which will increase the cost-effectiveness of robotic surgery for rectal resection.

Laparoscopic rectal resection requires a learning curve of 40–90 cases before a surgeon used to open surgery becomes proficient [11,70]. However, the learning curve for robotic resection is shorter for a surgeon with experience in open resection and requires only 21–43 cases to reach proficiency. This is probably due to the similarity in access, visualization, and maneuverability between the open and robotic approach [68,71,72]. This indicates that the robotic approach should be the method of choice for surgeons and surgical centers who are planning to change from open to minimal invasive rectal surgery as the learning curve is shorter. However, the higher costs should be considered. Centers performing laparoscopic rectal resection as routine can consider changing to the robotic approach if the costs are reasonable.

The main limitation of our meta-analysis was the overall quality of the evidence, which was judged as low and very low according to the GRADE assessment for the majority of outcomes. Most studies had low-quality or no blinding, which may have caused observer bias. In addition, the sample sizes were low for those outcomes that were not reported in all studies. There were also some outcomes with no events in either one or both treatment arms of some included studies and the meta-analyses may be underpowered to detect differences. Furthermore, due to the low number of studies which reported different types of rectal resection separately, we were not able to perform sub-analyses to compare different surgical methods in different procedures, such as low-anterior resections and ultra-low anterior resections, as well as in sphincter preserving surgeries versus abdominoperineal resections.

## 5. Conclusions

In conclusion, the robotic approach is a safe and effective method for rectal resection. It has considerable benefits over the open approach regarding surgical and histopathological outcomes; therefore, robot-assisted rectal surgery seems to be a reasonable substitution for open surgeries. When considering robotic surgery as a substitute for laparoscopic rectal surgery, the higher costs of the robotic approach are considerable, but robotic approach has lower rates of conversion and reoperation, less blood loss, and higher rate of negative CRM resection. With changes in the market space and introduction of more robotic systems in clinical use may bring down both the capital and instruments costs. This would lead to widespread adoption of the robotic surgery for rectal resections.

## Figures and Tables

**Table 1:** Study characteristics of the included studies in the qualitative analysis.

Study (Year)	Study Design	Country	Group	Age (Mean ± SD)	Sex (M/F)	BMI (Mean ± SD)	ASA (I/II/III/IV)	Tumor Location from Anal Verge, cm (Mean ± SD)	Robotic Surgical Technique	Sphincter-Saving Procedures	Follow-Up Duration, Months (Mean)
**Robotic vs. Open**											
Bertani et al. (2011) [46]	Prospective	Italy	ROB: 52	59.6 ± 11.6	31/21	24.8 ± 3.62	49 (I–II) 3 (III–V)	Median 8.4 (3–20)	Full-robotic	N/A	N/A
			Open: 34	63.2 ± 10.5	20/14	25.6 ± 3.85	28 (I–II) 6 (III–V)	Median 9.7 (3–25)		N/A	N/A
Somashekhar et al. (2015) [20]	RCT	India	ROB: 25	56.36 ± 8.21	17/8	31.51 ± 3.02	N/A	N/A	N/A	N/A	Median 5
			Open: 25	59.56 ± 5.75	15/10	29.84 ± 2.75	N/A	N/A		N/A	
Corbellini et al. (2016) [18]	Prospective	Italy	ROB: 65	64 (39–78)	35/30	36 (<25) 23 (25–30) 6 (>30)	N/A	Median 8 (1–12)	Hybrid	N/A	33 (1–57)
			Open: 55	62 (40–80)	36/19	33 (<25) 13 (25–30) 9 (>30)	N/A	Median 7 (1–12)		N/A	
Xu et al. (2017) [40]	RCT	China	ROB: 173	N/A	N/A	N/A	N/A	5	N/A	N/A	N/A
			Open: 154	N/A	N/A	N/A	N/A			N/A	N/A
ESCP (2018) [44]	Prospective	Denmark	ROB: 123	N/A	84/39	51 (<25) 45 (25–29.9) 26 (≥30)	89 (I–II) 34 (III–V)	N/A	N/A	**N/A**	N/A
			Open: 868	N/A	526/342	295 (<25) 357 (25–29.9) 201 (≥30)	516 (I–II) 348 (III–V)	N/A		**N/A**	N/A
**Robotic vs. Laparoscopic**											
Bailk et al. (2008) [6]	RCT	South Korea	ROB: 18	57.3 ± 6.3	14/4	22.8 ± 1.8	12/6/0/0	11.3 ± 2.5	Hybrid	18	N/A
			LAP: 18	62.0 ± 9.0	14/4	24.0 ± 2.5	10/6/1/1	1.0 ± 2.5		18	N/A
Patriti et al. (2009) [37]	RCT	Italy	ROB:29	68 ± 10	11/18	24 ± 6.2	2/13/14/0	5.9 ± 4.2	Hybrid	24	29.2
			LAP: 37	69 ± 10	12/25	25.4 ± 6.44	2/14/21/0	11 ± 4.5		34	18.7
Jimenez Rodrıguez et al. (2011) [36]	RCT	Spain	ROB: 28 *	68 ± 9.1	12/16	28.59 ± 2.5	14 (I–II) 14 (III)	4.8 ± 1.6	N/A	N/A	N/A
			LAP: 28 *	61.5 ± 15	17/11	26.75 ± 5.6	20 (I–II) 8 (III)	3.8 ± 0.7		N/A	N/A
J.Y. Kim et al. (2012) [50]	Prospective	South Korea	ROB: 30	54.13 ± 8.52	18/12	24.36 ± 2.44	29/1 (I/II)	(10–15 cm): 8 (6–10 cm): 16 (<6 cm): 6	Full-robotic	29	12
			LAP: 39	56.85 ± 11.14	20/19	24.01 ± 2.19	39/0 (I/II)	(10–15 cm): 14 (6–10 cm): 17 (<6 cm): 6		38	12
Corbellini et al. (2016) [18]	Prospective	Italy	ROB: 65	Median 64 (39–78)	35/30	36 (<25) 23 (25–30) 6 (>30)	N/A	Median 8 (1–12)	Hybrid	N/A	33 (1–57)
			LAP: 40	Median 64 (36–80)	23/17	17 (<25) 14 (25–30) 9 (>30)	N/A	Median 8.5 (1.2–12)		N/A	
H. J. Kim et al. (2017) [49]	Prospective	South Korea	ROB:130	60.5 ± 10.1	95/35	23.7 ± 3.2	71/48/11 (I/II/III)	5.9 ± 2.7	Hybrid	N/A	N/A
			LAP: 130	60 ± 9.3	95/35	23.3 ± 2.9	70/46/14 (I/II/III)	6.3 ± 2.6		N/A	N/A
Tolstrup et al. (2017) [38]	RCT	Denmark	ROB: 25	63 ± 10.9	18/7	27 ± 4.5	11/7/0/0	N/A	Full-robotic	3/10/3/9	N/A
			LAP: 26	68 ± 9.9	20/6	28 ± 4.3	10/8/1/0	N/A		4/11/5/6	N/A
Xu et al. (2017) [40]	RCT	China	ROB: 173	N/A	N/A	N/A	N/A	5	N/A	N/A	N/A
			LAP: 176	N/A	N/A	N/A	N/A			N/A	N/A
Wang et al. (2017) [39]	RCT	China	ROB: 71	Median 60.3 (36–68)	71/0	Median 22.9 (19.1–30.1)	N/A	25 (7–12) 46 (≤7)	N/A	69	12
			LAP: 66	Median 58.7 (36–71)	66/0	Median 22.4 (18.3–30.6)	N/A	26 (7–12 ) 40 (≤7)		63	12
Jayne et al. (2017) [19] AND Corrigan et al. (2018) [34] (ROLLAR trial)	RCT	UK	ROB: 237	64.4 ± 10.98	161/76	93 (<25) 90 (25–29.9) 54 (≥30)	39/150/46/0	71 (10–15) 107 (6–10) 57 (<6)	Hybrid+ Full- robotic	184	6
			LAP: 234	65.5 ± 11.93	159/75	87 (< 25) 92 (25–29.9) 55 (≥30)	52/124/52/1	69 (10–15) 99 (6–10) 61 (<6)		185	6
Debakey et al. (2018) [35]	RCT	Egypt	ROB: 21	Median 53.4 (32–67)	11/10	N/A	18/3/0/0	9 (10–15) 10 (5–10) 5 (<5):	Full-robotic	20	N/A
			LAP: 24	Median 50.3 (36–64)	13/11	N/A	18/6/0/0	13 (10–15) 8 (5–10) 3 (<5)		21	N/A
M. J. Kim et al. (2018) [17]	RCT	South Korea	ROB: 66	60.4 ± 9.7	51/15	24.1 ± 3.3	20/46 (I/II)	33 (>5) 33 (≤5)	Hybrid	65	N/A
			LAP: 73	59.7 ± 11.7	52/21	23.6 ± 3.0	30/43 (I/II)	38 (>5) 35 (≤5)		70	N/A
Asklid et al. (2018) [45]	Prospective	Sweden	ROB: 72	65.5 ± 10.4	43/29	24.6 ± 0.4	16/43/13/0	8.5 ± 3.6	N/A	N/A	N/A
			LAP: 47	70.1 ± 12	21/26	25.3 ± 0.5	8/24/14/1	9.8 ± 3.9		N/A	N/A
ESCP (2018) [44]	Prospective	Denmark	ROB: 123	N/A	84/39	51 (<25) 45 (25–29.9) 26 (≥30)	89 (I–II) 34 (III–V)	N/A	N/A	N/A	N/A
			LAP: 1176	N/A	727/449	361 (0–24.9) 504 (25–29.9)) 281 (≥30)	787 (I–II) 369 (III–V)	N/A		N/A	N/A
Galata et al. (2019) [48]	Prospective	Germany	ROB: 18	60.0 ± 11.8	10/8	26.0 ± 4.0	15/3 (II/III)	8.5 ± 4.0	Hybrid	N/A	12
			LAP: 33	62.3 ± 13.7	21/12	27.4 ± 5.5	21/12 (II/III)	7.7 ± 3.3		N/A	12
Polat et al. (2019) [52]	Prospective	Netherlands	ROB: 77	N/A	N/A	N/A	N/A	N/A	Full-robotic	N/A	N/A
			LAP: 34	N/A	N/A	N/A	N/A	N/A		N/A	N/A
Tang et al. (2020) [23]	RCT	China	ROB: 65	55.1 ± 12.1	36/29	22 ± 2.5	35/30/0/0	6 ± 2.4	Full-robotic	52	9–31
			LAP: 64	58.0 ± 9.7	36/28	22.1 ± 2.3	27/37/0/0	5.8 ± 2.6		44	9–31
Quijano et al. (2020) [53]	Prospective	Spain	ROB: 81	63.98 ± 9.68	54/37	25.98 ± 4.2	64 (I–II) 17 (III–V)	7.1	N/A	N/A	Median 12 (12–17)
			LAP: 104	61.37 ± 10.7	85/55	25.23 ± 5.38	91 (I–II) 13 (III–V)	7.3		N/A	Median 12 (12–17
Dulskas et al. (2021) [47]	Prospective	Lithuania	ROB: 62	Median 54 (35–74)	42/20	22.9 ± 3.0	N/A	22 (≥10 cm) 40 (6–10 cm)	N/A	N/A	N/A
			LAP: 78	Median 64 (26–89)	43/35	27.3 ± 3.3	N/A	74 (≥10 cm) 4 (6–10 cm)		N/A	N/A
Miura et al. (2022) [51]	Prospective	Japan	ROB: 35	Median 65 (37–75)	28/7	Median 22.9 (18.7–31.4)	33 (I–II) 2 (III–V)	Median 5.5 (2.5–8)	N/A	N/A	Median 22 (9–47)
			LAP: 14	Median 66 (34–77)	8/6	Median 21.4 (18.6–28.8)	13 (I–II) 1 (III–V)	Median 5.7 (3.5–8)		N/A	Median 33 (7–52)
REAL Trial, (2022) [41]	RCT	China	ROB: 586	59.1 ± 11	356/230	23.5 ± 3.3	554 (I–II) 32 (III)	5.9 ± 2.4	Full-robotic	444	30 (30–30)
			LAP: 585	60.7 ± 9.8	354/231	23.5 ± 3.1	558 (I–II) 27 (III)	5.8 ± 2.6		435	30 (30–30)
Feng et al. (2022) [42]	RCT	China	ROB: 174	58.2 ± 9.6	108/66	N/A	170 (I–II) 4 (III)	3.5 ± 0.7	Full-robotic	N/A	49 (38–62)
			LAP: 173	59.5 ± 10.2	113/60	N/A	166 (I–II) 7 (III)	3.6 ± 0.7		N/A	49 (38–62)
Liu et al. (2022) [43]	RCT	China	ROB: 47	58.7 ± 7.63	27/20	22.50 ± 1.67	46 (I–II) 1 (III)	5.15 ± 0.83	Full-robotic	N/A	12
			LAP: 45	58.53 ± 8.78	25/20	23.3 ± 2.37	45 (I–II) 0 (III)	5.23 ± 0.92		N/A	12

**Abbreviations:** SD: Standard deviation; M: Male; F: Female; BMI: Body mass index; ASA: American Society of Anesthesiologists score; cm: Centimeters; ROB: Robot-assisted surgery; N/A: Not available; RCT: Randomized controlled trial; LAP: Laparoscopy; ESCP: European Society of Coloproctology * This study includes 28 patients undergoing colorectal resection in each group, of them six patients in each group underwent rectal resection.

**Table 2:** Risk of bias assessment using the RoB2 tool for randomized controlled trials.

Study and Year	Domain 1	Domain 2	Domain 3	Domain 4	Domain 5	Domain 6	Domain 7	Overall
Bailk et al. (2008) [6]	Low	Low	Some concerns	Some concerns	Some concerns	Some concerns	Some concerns	Some concerns
Patriti et al. (2009) [37]	Some concerns	Some concerns	Some concerns	Some concerns	Some concerns	Some concerns	Some concerns	Some concerns
Jimenez Rodrıguez et al. (2011) [36]	Low	Some concerns	Some concerns	Some concerns	Low	Low	Low	Some concerns
Somashekhar et al. (2015) [20]	Some concerns	Some concerns	High	High	Some concerns	Some concerns	High	High
Jayne et al. (2017) [19]	Low	Some concerns	Some concerns	Low	Low	Low	Low	Some concerns
Xu et al. (2017) [40]	Some concerns	Some concerns	Some concerns	Some concerns	Some concerns	Low	Some concerns	Some concerns
Wang et al. (2017) [39]	Low	Low	Some concerns	Some concerns	Some concerns	Some concerns	Some concerns	Some concerns
Tolstrup et al. (2017) [38]	Low	Some concerns	High	High	Low	Some concerns	Low	High
Corrigan et al. (2018) [34]	Low	Some concerns	Some concerns	Low	Low	Low	Low	Some concerns
M. J. Kim et al. (2018) [17]	Low	Low	High	Low	Low	Low	Low	High
Debakey et al. (2018) [35]	Low	High	High	High	Low	Some concerns	Some concerns	High
Tang et al. (2020) [23]	Low	Low	Some concerns	Some concerns	Some concerns	Some concerns	Some concerns	Some concerns
REAL Trial, (2022) [41]	Low	Low	High	Low	Low	Low	Low	High
Feng et al. (2022) [42]	Low	Some concerns	High	Low	Low	Low	Low	High
Liu et al. (2022) [43]	High	High	High	Some concerns	Some concerns	Low	Some concerns	High

Domain 1: Random sequence generation; Domain 2: Allocation concealment; Domain 3: Blinding of participants and personnel; Domain 4: Blinding of outcome assessment; Domain 5: Incomplete outcome data; Domain 6: Selective reporting; Domain 7: Other bias.

**Table 3:** Risk of bias assessment using ROBINS-I for non-randomized prospective studies.

Study and Year	Domain 1	Domain 2	Domain 3	Domain 4	Domain 5	Domain 6	Domain 7	Overall
Bertani et al. (2011) [46]	Moderate	Low	Low	Low	Moderate	Low	Moderate	Moderate
J.Y. Kim et al. (2012) [50]	Serious	Moderate	Low	Low	Moderate	Low	Moderate	Serious
ESCP (2018) [44]	Moderate	Low	Low	Moderate	Moderate	Low	Low	Moderate
Corbellini et al. (2016) [18]	Moderate	Moderate	Moderate	Moderate	Moderate	Low	Moderate	Moderate
H. J. Kim et al. (2017) [49]	Moderate	Moderate	Low	Low	Serious	Moderate	Moderate	Serious
Asklid et al. (2018) [45]	Moderate	Moderate	Low	Low	Moderate	Low	Moderate	Moderate
Galata et al. (2019) [48]	Moderate	Moderate	Low	Low	Moderate	Low	Moderate	Moderate
Polat et al. (2019) [52]	Moderate	Moderate	Low	Low	Moderate	Low	Moderate	Moderate
Quijano et al. (2020) [53]	Moderate	Moderate	Moderate	Low	Moderate	Moderate	Low	Moderate
Dulskas et al. (2021) [47]	Serious	Moderate	Moderate	Moderate	Moderate	Moderate	Moderate	Serious
Miura et al. (2022) [51]	Moderate	Moderate	Moderate	Moderate	Serious	Moderate	Serious	Serious

Domain 1: Bias due to confounding; Domain 2: Bias in selection of participants into the study; Domain 3: Bias in classification of interventions; Domain 4: Bias due to deviations from intended interventions; Domain 5: Bias due to missing data; Domain 6: Bias in measurements of outcomes; Domain 7: Bias in selection of the reported result.

## Data Availability

The data presented in this study are available on request from the corresponding author.

## Supplementary Materials

The following supporting information can be downloaded at: https://www.mdpi.com/article/10.3390/cancers15030839/s1, Table S1. Grades of recommendation, assessment, development, and evaluation (GRADE) assessment for outcomes of interest. Table S2. Meta analyses results and quality of evidence for outcome, Figure S1. Forest plots comparing 1-year recurrence free survival between robotic and laparoscopic rectal resection (blue boxes representing odd ratios, arrows representing 95% confidence intervals, and diamonds representing point estimates of pooled odd ratios or mean differences). Figure S2. Forest plots comparing 3-year recurrence free survival between robotic and laparoscopic rectal resection (blue boxes representing odd ratios, arrows representing 95% confidence intervals, and diamonds representing point estimates of pooled odd ratios or mean differences). Figure S3. Forest plots comparing 1-year overall survival between robotic and laparoscopic rectal resection (blue boxes representing odd ratios, arrows representing 95% confidence intervals, and diamonds representing point estimates of pooled odd ratios or mean differences). Figure S4. Forest plots comparing 3-year overall survival between robotic and laparoscopic rectal resection (blue boxes representing odd ratios, arrows representing 95% confidence intervals, and diamonds representing point estimates of pooled odd ratios or mean differences). Figure S5. Forest plot comparing proximal resection margin between robotic and laparoscopic rectal resection (green boxes representing mean differences, arrows representing 95% confidence intervals, and diamonds representing point estimates of pooled odd ratios or mean differences). Figure S6. Forest plot comparing distal resection margin between robotic and laparoscopic rectal resection (green boxes representing mean differences, arrows representing 95% confidence intervals, and diamonds representing point estimates of pooled odd ratios or mean differences). Figure S7. Forest plot comparing ileus between robotic and laparoscopic rectal resection (blue boxes representing odd ratios, arrows representing 95% confidence intervals, and diamonds representing point estimates of pooled odd ratios or mean differences). Figure S8. Forest plot comparing pain score between robotic and laparoscopic rectal resection (green boxes representing mean differences, arrows representing 95% confidence intervals, and diamonds representing point estimates of pooled odd ratios or mean differences). Figure S9. Forest plot comparing respiratory complications between robotic and laparoscopic rectal resection (blue boxes representing odd ratios, arrows representing 95% confidence intervals, and diamonds representing point estimates of pooled odd ratios or mean differences). Figure S10. Forest plot comparing urinary complications between robotic and laparoscopic rectal resection (blue boxes representing odd ratios, arrows representing 95% confidence intervals, and diamonds representing point estimates of pooled odd ratios or mean differences).
