# Supplementary material for: Outcomes of Robot-Assisted Surgery in Rectal Cancer Compared with Open and Laparoscopic Surgery
Source: Cancers (Basel). 2023 Jan 29;15(3):839. doi: 10.3390/cancers15030839 (PMC9913667; doi:10.3390/cancers15030839)
Supplement: Supplementary file 1 [file cancers-15-00839-s001.zip › cancers-2144743-supplementary.pdf]

Results

Robotic surgery vs laparoscopic surgery

Proximal resection margin

The proximal resection margin was reported in seven studies, comprising 2,008 patients. The pooled analysis showed no significant differences in the proximal resection margin between the robotic surgery and laparoscopic surgery group (MD=-0.19; 95% CI: -0.47– 0.85; *p*=0.57). There was high heterogeneity between studies in this analysis (I^2=97%; *p*<0.00001) (Supplementary Figure S5).

Distal resection margin

The distal resection margin was reported in nine studies, comprising 1,790 patients. The pooled analysis showed no significant differences in the distal resection margin between the robotic surgery group and laparoscopic surgery group (MD=0.04; 95% CI: -0.07–0.15; *p*=0.45). There was no relevant heterogeneity between studies in this analysis (I^2=19%; *p*=0.27). (Supplementary Figure S6)

Ileus

Ileus was reported in nine studies, enrolling 2,690 patients. The pooled analysis showed no significant difference in ileus between the robotic surgery arm and laparoscopic surgery arm (OR=1.41; 95% CI: 0.92–2.16; *p*=0.12). No heterogeneity was detected between studies in this meta-analysis (I^2=1%; *p*=0.43) (Supplementary Figure S7).

Pain score

The VAS pain score was reported in two studies. The meta-analysis showed no significant differences in VAS scores between the two arms (MD=-0.06; 95% CI: -0.36–0.23; *p*=0.67).

No heterogeneity was detected between studies in this analysis (I^2=0%; *p*=0.75) (Supplementary Figure S8).

Respiratory complications

Respiratory complications were reported in five studies, enrolling 2,177 patients. The pooled analysis showed no difference in respiratory complications between the robotic surgery group and the laparoscopic surgery group (OR=1.45; 95% CI: 0.72–2.93; *p*=0.30). There was no heterogeneity between studies in this analysis (I^2=0%; *p*=0.44) (Supplementary Figure S9).

Urinary complications

Urinary complications were reported in nine studies comprising 2,771 patients, 1,383 of whom underwent robotic surgery and 1,388 of whom underwent laparoscopic surgery. Urinary complications were reported in 54/1,388 (3.9%) patients in the robotic surgery group and in 44/1,383 (3.2%) patients in the laparoscopic surgery group. This difference was not significant (OR=1.22; 95% CI: 0.81–1.86; *p*=0.34). No heterogeneity was detected in this analysis (I^2=0%; *p*=0.81) (Supplementary Figure S10).

Supplementary Table S1. Grades of recommendation, assessment, development, and evaluation (GRADE) assessment for outcomes of interest.									
**Comparison**	**Studies**	**GRADE**							
		**Risk of bias**	**Inconsistency**	**Indirectness**	**Imprecision**	**Publication bias**	**Large or moderate effect size**	**Exposure-response gradient**	**Overall quality**
**Robotic vs Open**									
Intraoperative complications	4	Serious	Not serious	Not serious	Serious	Not serious	Not present	Not present	Low
Estimated blood loss	4	Serious	Serious	Not serious	Serious	Not serious	Not present	Not present	Very low
Operation time	3	Serious	Serious	Not serious	Serious	Not serious	Not present	Not present	Very low
Anastomotic leakage	4	Serious	Not serious	Not serious	Serious	Not serious	Not present	Not present	Very low
Surgical site infection	3	Serious	Not serious	Not serious	Serious	Not serious	Not present	Not present	Low
Postoperative complications	5	Serious	Serious	Not serious	Serious	Not serious	Not present	Not present	Very low
Major complications	3	Serious	Not serious	Not serious	Serious	Not serious	Not present	Not present	Low
Length of hospital stay	5	Serious	Serious	Not serious	Serious	Not serious	Not present	Not present	Very low
Total harvested lymph nodes	4	Serious	Not serious	Not serious	Serious	Not serious	Not present	Not present	Low
Microscopic margin-free resection	3	Serious	Not serious	Not serious	Serious	Not serious	Not present	Not present	Low
Circumferential resection margin	4	Serious	Not serious	Not serious	Serious	Not serious	Not present	Not present	Very low
**Robotic vs Laparoscopic**									
Intraoperative complications	8	Serious	Serious	Not serious	Serious	Not serious	Not present	Not present	Very low
Conversion to open surgery	17	Serious	Serious	Not serious	Not serious	Not serious	Not present	Not present	Low
Estimated blood loss	9	Serious	Serious	Not serious	Not serious	Not serious	Not present	Not present	Low
Operation time	19	Serious	Serious	Not serious	Not serious	Not serious	Not present	Not present	Low
Anastomotic leakage	14	Serious	Not serious	Not serious	Serious	Not serious	Not present	Not present	Very low
Surgical site complications	12	Serious	Not serious	Not serious	Serious	Not serious	Not present	Not present	Low
Postoperative complications	18	Serious	Not serious	Not serious	Serious	Not serious	Not present	Not present	Low
Major complications	15	Serious	Not serious	Not serious	Serious	Not serious	Not present	Not present	Low
Reoperation	12	Serious	Not serious	Not serious	Not serious	Not serious	Not present	Not present	Moderate
Length of hospital stay	18	Serious	Serious	Not serious	Serious	Not serious	Not present	Not present	Very low
TME completeness	11	Serious	Not serious	Not serious	Serious	Not serious	Not present	Not present	Low
Total harvested lymph nodes	15	Serious	Serious	Not serious	Serious	Not serious	Not present	Not present	Very low
Microscopic margin-free resection	8	Serious	Not serious	Not serious	Serious	Not serious	Not present	Not present	Very low
Circumferential resection margin	14	Serious	Not serious	Not serious	Not serious	Not serious	Not present	Not present	Moderate
Up to 90 days mortality	14	Serious	Not serious	Not serious	Serious	Not serious	Not present	Not present	Low
1-year recurrence free survival	3	Serious	Not serious	Not serious	Serious	Not serious	Not present	Not present	Low
3-year recurrence free survival	3	Serious	Serious	Not serious	Serious	Not serious	Not present	Not present	Very low
1-year overall survival	3	Serious	Not serious	Not serious	Serious	Not serious	Not present	Not present	Very low

3-year overall survival	3	Serious	Not serious	Not serious	Serious	Not serious	Not present	Not present	Very low
Operative cost	2	Serious	Not serious	Not serious	Serious	Not serious	Not present	Not present	Low
Proximal resection margin	7	Serious	Not serious	Serious	Serious	Not serious	Not present	Not present	Very low
Distal resection margin	9	Serious	Not serious	Not serious	Serious	Not serious	Not present	Not present	Low
Ileus	9	Very serious	Not serious	Not serious	Serious	Not serious	Not present	Not present	Very low
Pain score	2	Serious	Not serious	Not serious	Serious	Not serious	Not present	Not present	Very low
Respiratory complications	5	Serious	Not serious	Not serious	Serious	Not serious	Not present	Not present	Low
Urinary complications	9	Serious	Not serious	Not serious	Serious	Not serious	Not present	Not present	Low

Abbreviation: TME, Total Mesorectal Excision

Supplementary Table S2. Meta analyses results and quality of evidence for outcome				
**Outcome**	**Number of studies**	**Pooled relative effect (95% CI)**	**Statistical heterogeneity: I^2 (%); *p*-value**	**Quality of evidence (GRADE)**
**Robotic vs Open**				
Intraoperative complications	4	OR:1.21; (0.42 – 3.48)	0; 0.56	Low
Estimated blood loss	4	MD:156.63; (62.36 – 250.91)	94; <0.00001	Very low
Operation time	3	MD:-66.90; (-93.35 – -40.46)	83; 0.003	Very low
Anastomotic leakage	4	OR:1.04; (0.55 – 1.99)	0; 0.59	Very low
Surgical site infection	3	OR:4.49; (1.05 – 19.24)	0; 0.72	Low
Postoperative complications	5	OR:1.33; (0.65 – 2.71)	76; 0.002	Very low
Major complications	3	OR:0.91; (0.56 – 1.47)	2; 0.36	Low
Length of hospital stay	5	MD:2.51; (0.35 – 4.67)	94; <0.0001	Very low
Total harvested lymph nodes	4	MD:0.86; (0.14 – 1.59)	27; 0.25	Low
Microscopic margin-free resection	3	OR:6.01; (1.13 – 31.91)	0; 0.99	Low
Circumferential resection margin	4	OR:3.39; (1.11 – 10.40)	0; 0.79	Very low
**Robotic vs Laparoscopic**				
Intraoperative complications	8	OR:1.48; (0.95 – 2.32)	35; 0.09	Very low
Estimated blood loss	9	MD:20.47; (7.57 – 33.36)	99; <0.00001	Low
Conversion to open surgery	17	OR:3.13; (1.87 – 5.21)	33; 0.10	Low
Operation time	19	MD: -36.29 ; (-47.34– -25.25)	98; <0.00001	Low
Anastomotic leakage	14	OR:1.22; (0.90 – 1.65)	0; 0.54	Very low
Surgical site complications	12	OR:1.04; (0.63 – 1.73)	0; 0.90	Low
Postoperative complications	18	OR:1.11; (0.86 – 1.43)	43; 0.03	Low
Major complications	15	OR:1.19; (0.84 – 1.69)	15; 0.29	Low
Reoperation	12	OR:1.69; (1.10 – 2.62)	0; 0.95	Moderate
Length of hospital stay	18	MD:-0.00; (-0.55 – 0.54)	96; <0.00001	Very low
TME completeness	11	OR:1.09; (0.74 – 1.60)	29; 0.18	Low
Total harvested lymph nodes	15	MD:0.38; (-0.39 – 1.16)	59; 0.002	Very low
Microscopic margin-free resection	8	OR:0.99; (0.36 – 2.70)	0; 0.50	Very low
Circumferential resection margin	14	OR:1.56; (1.11 – 2.20)	0; 0.59	Moderate
Up to 90 days mortality	14	OR:1.13; (0.30 – 4.20)	0; 0.95	Low
1-year recurrence free survival	3	OR:0.68; (0.28 – 1.62)	0; 0.82	Low
3-year recurrence free survival	3	OR:1.08; (0.39 – 2.96)	52; 0.12	Very low
1-year overall survival	3	OR:2.10; (0.54 – 8.07)	0; 0.64	Very low
3-year overall survival	3	OR: 1.13; (0.32 – 4.01)	48; 0.14	Very low
Operative cost (numbers are given in $1000)	2	MD:-0.83; (-1.40 – -0.27)	99; <0.00001	Low
Proximal resection margin	7	MD:-0.19; (-0.47 – 0.85)	97; <0.00001	Very low
Distal resection margin	9	MD:0.04; (-0.07 – 0.15)	19; 0.27	Low

Ileus	9	OR:1.41; (0.92 – 2.16)	1; 0.43	Very low
Pain score	2	MD:-0.06; (-0.36 – 0.23)	0; 0.75	Very low
Respiratory complications	5	OR=1.45; (0.72–2.93)	0; 0.44	Low
Urinary complications	9	OR=1.22; (0.81–1.86)	0; 0.81	Low
**Abbreviation:** TME: Total mesorectal excision, OR: odds ratio, MD: mean difference, CI: confidence interval				

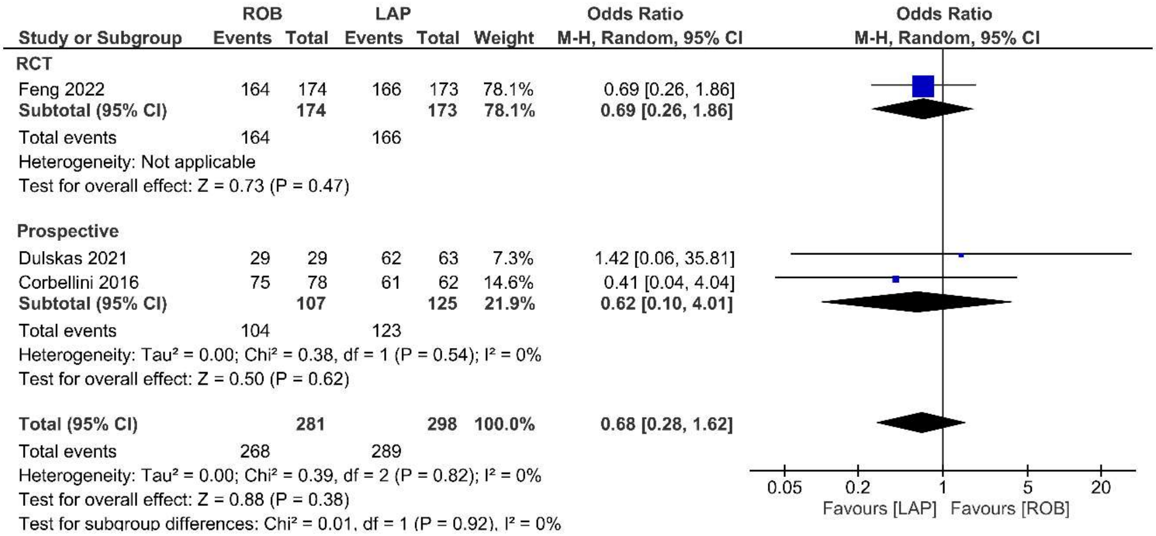

Supplementary Figure S1. Forest plots comparing 1-year recurrence free survival between robotic and laparoscopic rectal resection (blue boxes representing odd ratios, arrows representing 95% confidence intervals, and diamonds representing point estimates of pooled odd ratios or mean differences).

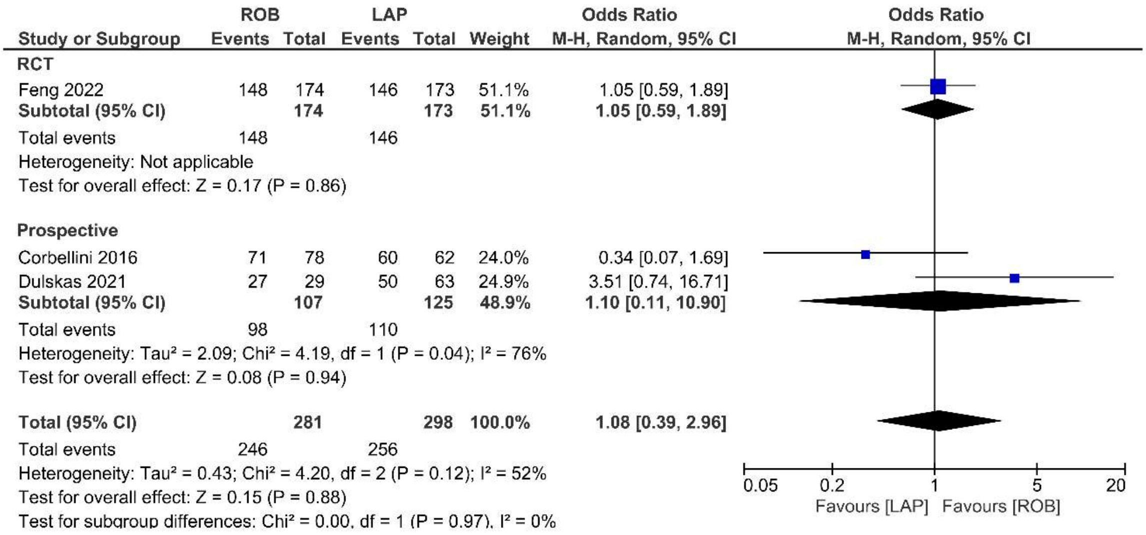

Supplementary Figure S2. Forest plots comparing 3-year recurrence free survival between robotic and laparoscopic rectal resection (blue boxes representing odd ratios, arrows representing 95% confidence intervals, and diamonds representing point estimates of pooled odd ratios or mean differences).

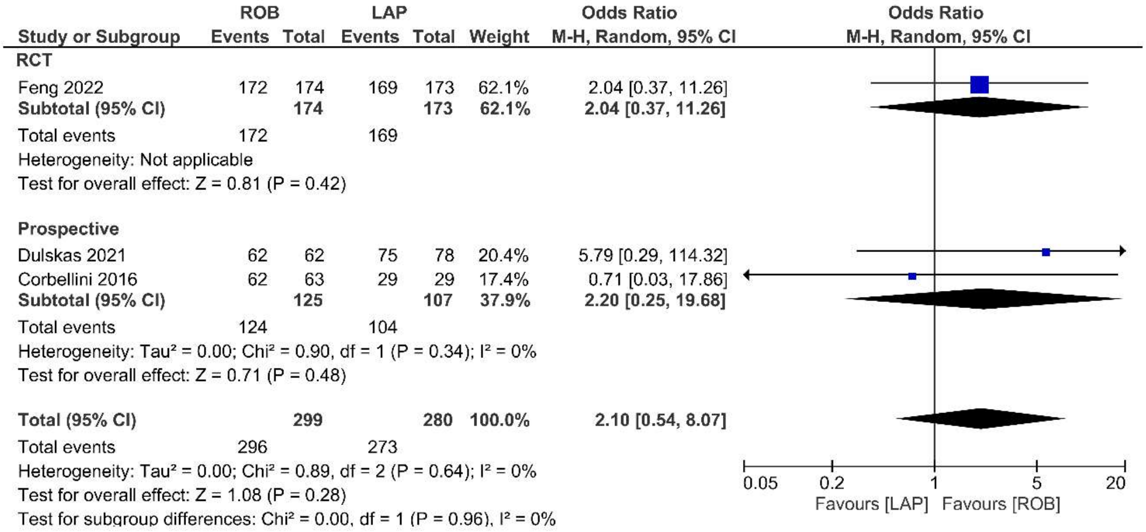

Supplementary Figure S3. Forest plots comparing 1-year overall survival between robotic and laparoscopic rectal resection (blue boxes representing odd ratios, arrows representing 95% confidence intervals, and diamonds representing point estimates of pooled odd ratios or mean differences).

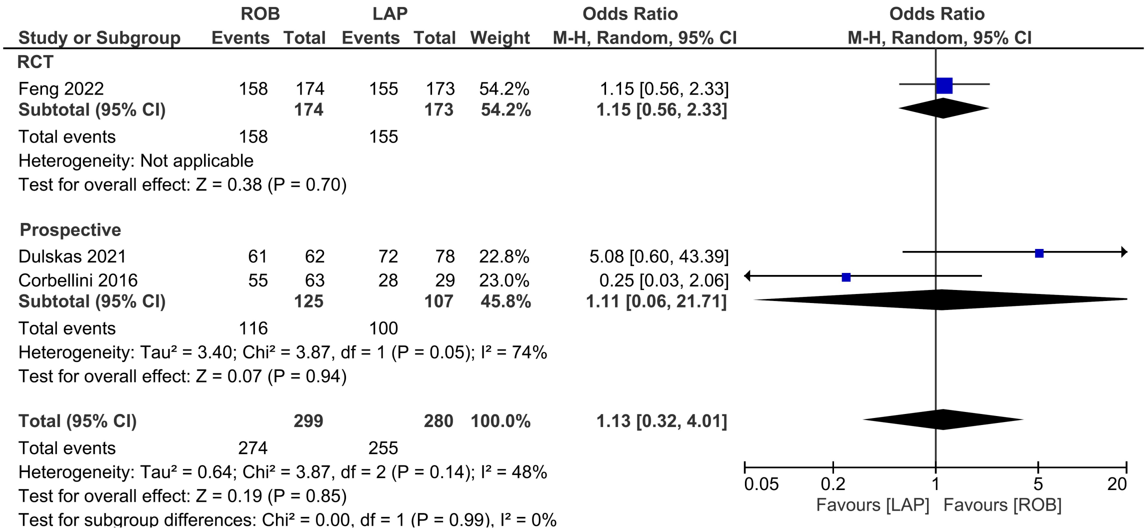

Supplementary Figure S4. Forest plots comparing 3-year overall survival between robotic and laparoscopic rectal resection (blue boxes representing odd ratios, arrows representing 95% confidence intervals, and diamonds representing point estimates of pooled odd ratios or mean differences).

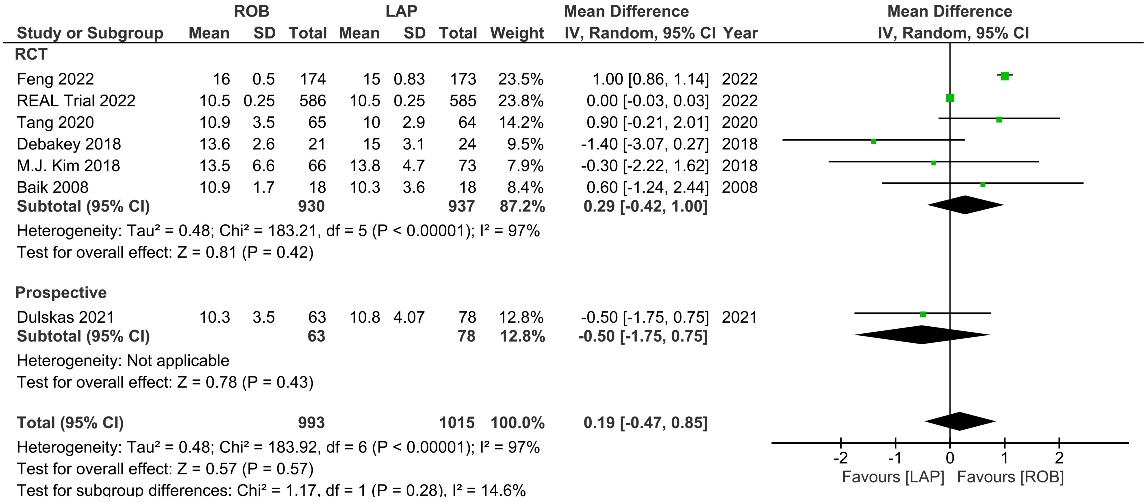

Supplementary Figure S5. Forest plot comparing proximal resection margin between robotic and laparoscopic rectal resection (green boxes representing mean differences, arrows representing 95% confidence intervals, and diamonds representing point estimates of pooled odd ratios or mean differences).

Study or Subgroup	ROB Mean	ROB SD	ROB Total	LAP Mean	LAP SD	LAP Total	Weight	Mean Difference IV, Random, 95% CI	Year
**RCT**									
REAL Trial 2022	2	0.25	586	2	0.25	585	54.5%	0.00 [-0.03, 0.03]	2022
Tang 2020	2.6	0.7	65	2.5	0.6	64	16.4%	0.10 [-0.12, 0.32]	2020
Debakey 2018	2.3	0.7	21	2.1	0.5	24	7.7%	0.20 [-0.16, 0.56]	2018
M.J. Kim 2018	1.3	1.4	66	1.2	1.1	73	5.9%	0.10 [-0.32, 0.52]	2018
Jimenez Rodriguez 2011	4.8	1.6	6	3.8	0.7	6	0.6%	1.00 [-0.40, 2.40]	2011
Baik 2008	4	1.1	18	3.7	1.5	18	1.5%	0.30 [-0.56, 1.16]	2008
**Subtotal (95% CI)**			**762**			**770**	**86.6%**	**0.00 [-0.02, 0.03]**	
Heterogeneity: Tau² = 0.00; Chi² = 4.52, df = 5 (P = 0.48); I² = 0%									
Test for overall effect: Z = 0.28 (P = 0.78)									
**Prospective**									
Miura 2022	1.5	0.62	35	1.87	0.72	14	5.7%	-0.37 [-0.80, 0.06]	2022
Dulskas 2021	3.1	1.5	62	2.7	1.5	78	4.3%	0.40 [-0.10, 0.90]	2021
J.Y. Kim 2012	2.79	1.02	30	2.86	1.36	39	3.5%	-0.07 [-0.63, 0.49]	2012
**Subtotal (95% CI)**			**127**			**131**	**13.4%**	**-0.02 [-0.49, 0.44]**	
Heterogeneity: Tau² = 0.10; Chi² = 5.25, df = 2 (P = 0.07); I² = 62%									
Test for overall effect: Z = 0.11 (P = 0.92)									
**Total (95% CI)**			**889**			**901**	**100.0%**	**0.04 [-0.07, 0.15]**	
Heterogeneity: Tau² = 0.01; Chi² = 9.91, df = 8 (P = 0.27); I² = 19%									
Test for overall effect: Z = 0.76 (P = 0.45)									
Test for subgroup differences: Chi² = 0.02, df = 1 (P = 0.90), I² = 0%									

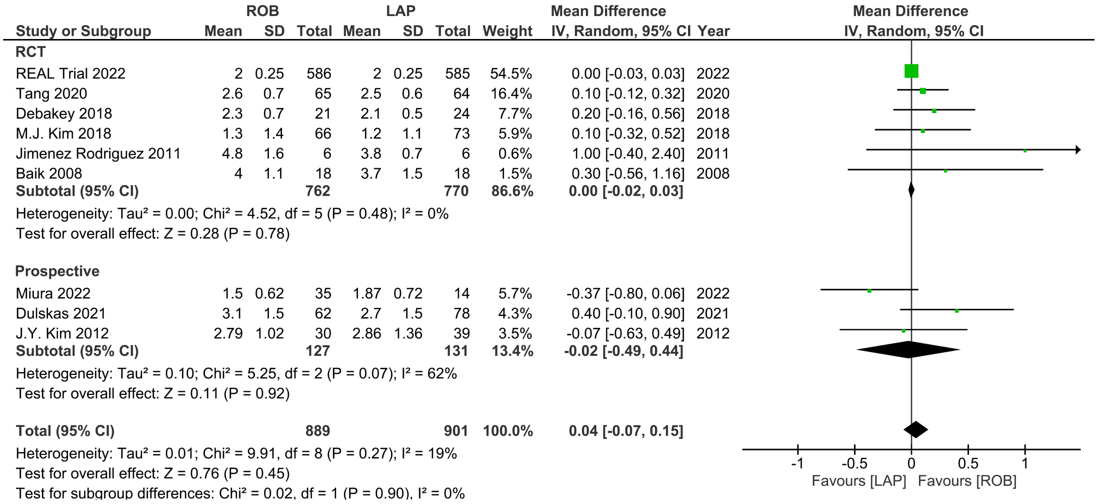

Supplementary Figure S6. Forest plot comparing distal resection margin between robotic and laparoscopic rectal resection (green boxes representing mean differences, arrows representing 95% confidence intervals, and diamonds representing point estimates of pooled odd ratios or mean differences).

Study or Subgroup	LAP Events	LAP Total	ROB Events	ROB Total	Weight	Odds Ratio M-H, Random, 95% CI	Year
**RCT**							
Feng 2022	6	173	4	174	11.1%	1.53 [0.42, 5.51]	2022
REAL Trial 2022	11	585	5	586	16.1%	2.23 [0.77, 6.45]	2022
Debakey 2018	3	24	2	21	5.1%	1.36 [0.20, 9.02]	2018
M.J. Kim 2018	9	73	6	66	15.3%	1.41 [0.47, 4.19]	2018
ROLARR Trial 2018	22	234	13	237	35.6%	1.79 [0.88, 3.64]	2018
Patriti 2009	1	37	2	29	3.0%	0.38 [0.03, 4.35]	2009
**Subtotal (95% CI)**		**1126**		**1113**	**86.1%**	**1.63 [1.03, 2.58]**	
Total events	52		32				
Heterogeneity: Tau² = 0.00; Chi² = 1.89, df = 5 (P = 0.86); I² = 0%							
Test for overall effect: Z = 2.09 (P = 0.04)							
**Prospective**							
Dulskas 2021	0	78	5	62	2.2%	0.07 [0.00, 1.23]	2021
Galata 2019	3	33	3	18	6.2%	0.50 [0.09, 2.78]	2019
H.J. Kim 2017	3	130	2	130	5.6%	1.51 [0.25, 9.20]	2017
**Subtotal (95% CI)**		**241**		**210**	**13.9%**	**0.50 [0.11, 2.37]**	
Total events	6		10				
Heterogeneity: Tau² = 0.78; Chi² = 3.42, df = 2 (P = 0.18); I² = 41%							
Test for overall effect: Z = 0.87 (P = 0.38)							
**Total (95% CI)**		**1367**		**1323**	**100.0%**	**1.41 [0.92, 2.16]**	
Total events	58		42				
Heterogeneity: Tau² = 0.00; Chi² = 8.04, df = 8 (P = 0.43); I² = 1%							
Test for overall effect: Z = 1.56 (P = 0.12)							
Test for subgroup differences: Chi² = 2.03, df = 1 (P = 0.15), I² = 50.9%							

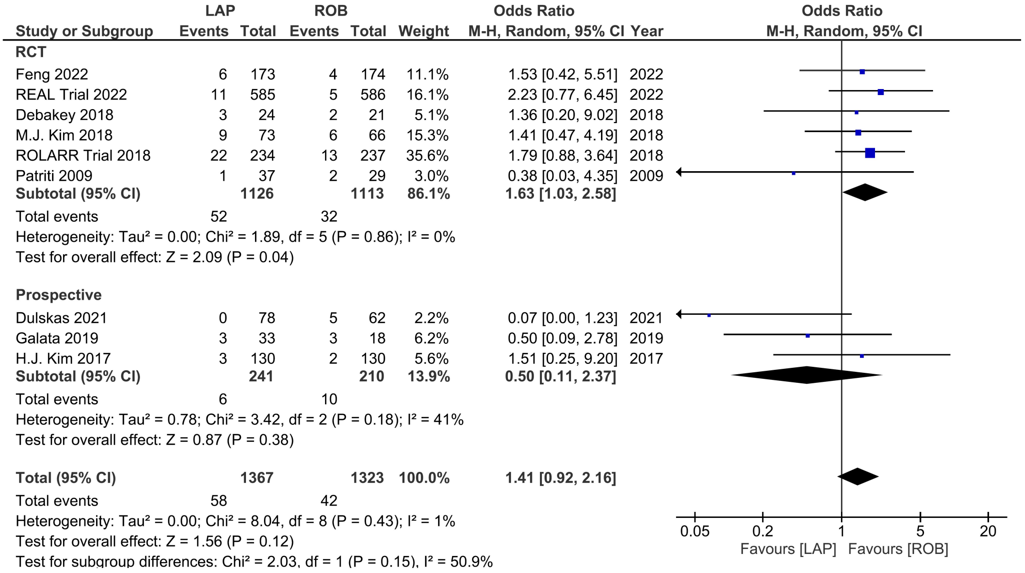

Supplementary Figure S7. Forest plot comparing ileus between robotic and laparoscopic rectal resection (blue boxes representing odd ratios, arrows representing 95% confidence intervals, and diamonds representing point estimates of pooled odd ratios or mean differences).

Study or Subgroup	LAP Mean	LAP SD	LAP Total	ROB Mean	ROB SD	ROB Total	Weight	Mean Difference IV, Random, 95% CI
**RCT**								
Jimenez Rodriguez 2011	1.7	0.7	28	1.8	0.7	28	64.6%	-0.10 [-0.47, 0.27]
**Subtotal (95% CI)**			**28**			**28**	**64.6%**	**-0.10 [-0.47, 0.27]**
Heterogeneity: Not applicable								
Test for overall effect: Z = 0.53 (P = 0.59)								
**Prospective**								
Galata 2019	1.1	1.1	33	1.1	0.7	18	35.4%	0.00 [-0.50, 0.50]
**Subtotal (95% CI)**			**33**			**18**	**35.4%**	**0.00 [-0.50, 0.50]**
Heterogeneity: Not applicable								
Test for overall effect: Z = 0.00 (P = 1.00)								
**Total (95% CI)**			**61**			**46**	**100.0%**	**-0.06 [-0.36, 0.23]**
Heterogeneity: Tau² = 0.00; Chi² = 0.10, df = 1 (P = 0.75); I² = 0%								
Test for overall effect: Z = 0.43 (P = 0.67)								
Test for subgroup differences: Chi² = 0.10, df = 1 (P = 0.75), I² = 0%								

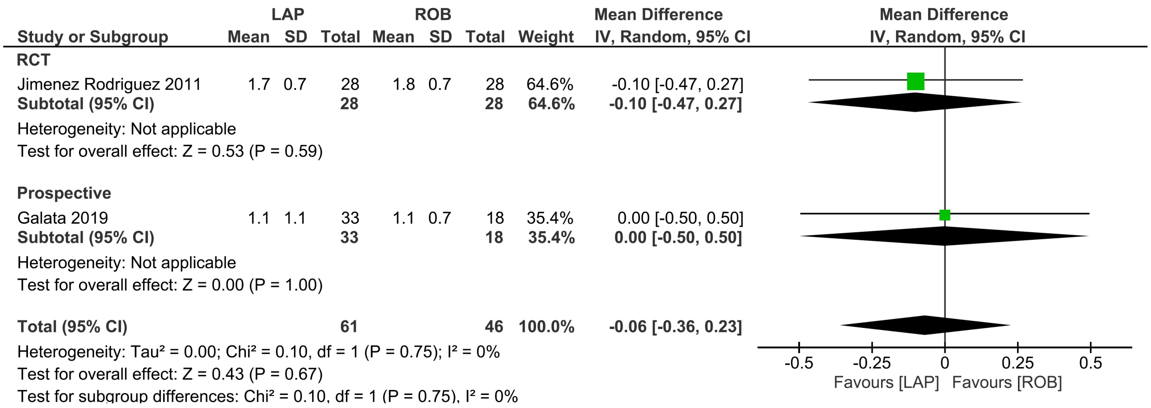

Supplementary Figure S8. Forest plot comparing pain score between robotic and laparoscopic rectal resection (green boxes representing mean differences, arrows representing 95% confidence intervals, and diamonds representing point estimates of pooled odd ratios or mean differences).

Study or Subgroup	LAP Events	LAP Total	ROB Events	ROB Total	Weight	Odds Ratio M-H, Random, 95% CI
**RCT**						
Feng 2022	4	173	2	174	16.9%	2.04 [0.37, 11.26]
REAL Trial 2022	7	585	4	586	32.4%	1.76 [0.51, 6.05]
ROLARR Trial 2018	6	234	4	237	30.2%	1.53 [0.43, 5.50]
Tolstrup 2017	0	26	4	25	5.6%	0.09 [0.00, 1.77]
Wang 2017	3	66	2	71	14.9%	1.64 [0.27, 10.15]
**Subtotal (95% CI)**		**1084**		**1093**	**100.0%**	**1.45 [0.72, 2.93]**
Total events	20		16			
Heterogeneity: Tau² = 0.00; Chi² = 3.77, df = 4 (P = 0.44); I² = 0%						
Test for overall effect: Z = 1.04 (P = 0.30)						
**Total (95% CI)**		**1084**		**1093**	**100.0%**	**1.45 [0.72, 2.93]**
Total events	20		16			
Heterogeneity: Tau² = 0.00; Chi² = 3.77, df = 4 (P = 0.44); I² = 0%						
Test for overall effect: Z = 1.04 (P = 0.30)						
Test for subgroup differences: Not applicable						

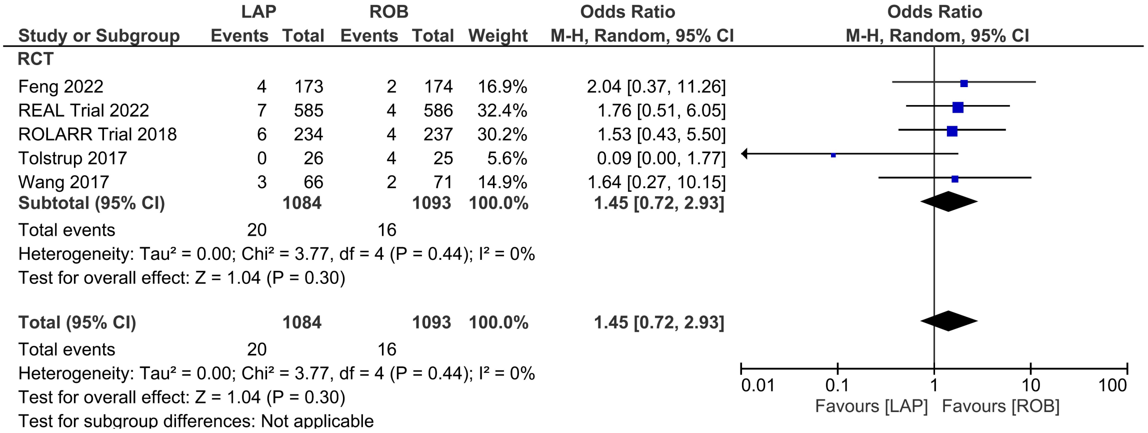

Supplementary Figure S9. Forest plot comparing respiratory complications between robotic and laparoscopic rectal resection (blue boxes representing odd ratios, arrows representing 95% confidence intervals, and diamonds representing point estimates of pooled odd ratios or mean differences).

Study or Subgroup	LAP Events	LAP Total	ROB Events	ROB Total	Weight	Odds Ratio M-H, Random, 95% CI	Year
**RCT**							
Feng 2022	13	173	7	174	19.4%	1.94 [0.75, 4.98]	2022
REAL Trial 2022	17	585	10	586	27.8%	1.72 [0.78, 3.80]	2022
Tang 2020	2	64	1	65	2.9%	2.06 [0.18, 23.35]	2020
ROLARR Trial 2018	14	234	17	237	32.3%	0.82 [0.40, 1.71]	2018
M.J. Kim 2018	3	73	5	66	8.0%	0.52 [0.12, 2.28]	2018
Tolstrup 2017	1	26	1	25	2.2%	0.96 [0.06, 16.23]	2017
Wang 2017	1	66	1	71	2.2%	1.08 [0.07, 17.57]	2017
Patriti 2009	1	37	1	29	2.2%	0.78 [0.05, 12.99]	2009
**Subtotal (95% CI)**		**1258**		**1253**	**97.0%**	**1.21 [0.79, 1.84]**	
Total events	52		43				
Heterogeneity: Tau² = 0.00; Chi² = 4.35, df = 7 (P = 0.74); I² = 0%							
Test for overall effect: Z = 0.87 (P = 0.38)							
**Prospective**							
H.J. Kim 2017	2	130	1	130	3.0%	2.02 [0.18, 22.51]	2017
**Subtotal (95% CI)**		**130**		**130**	**3.0%**	**2.02 [0.18, 22.51]**	
Total events	2		1				
Heterogeneity: Not applicable							
Test for overall effect: Z = 0.57 (P = 0.57)							
**Total (95% CI)**		**1388**		**1383**	**100.0%**	**1.22 [0.81, 1.86]**	
Total events	54		44				
Heterogeneity: Tau² = 0.00; Chi² = 4.52, df = 8 (P = 0.81); I² = 0%							
Test for overall effect: Z = 0.95 (P = 0.34)							
Test for subgroup differences: Chi² = 0.17, df = 1 (P = 0.68), I² = 0%							

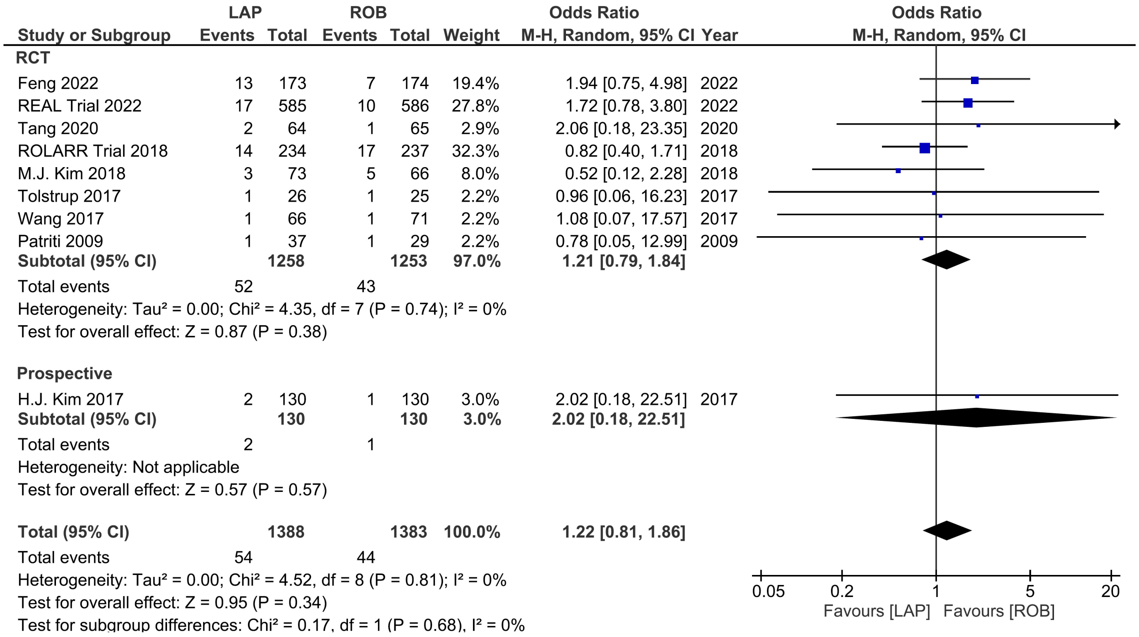

Supplementary Figure S10. Forest plot comparing urinary complications between robotic and laparoscopic rectal resection (blue boxes representing odd ratios, arrows representing 95% confidence intervals, and diamonds representing point estimates of pooled odd ratios or mean differences).
